# A Hormone-Responsive C1-Domain-Containing Protein At5g17960 Mediates Stress Response in *Arabidopsis thaliana*

**DOI:** 10.1371/journal.pone.0115418

**Published:** 2015-01-15

**Authors:** Ravindran Vijay Bhaskar, Bijayalaxmi Mohanty, Vivek Verma, Edward Wijaya, Prakash P. Kumar

**Affiliations:** 1 Department of Biological Sciences, Faculty of Science, National University of Singapore, Singapore, Singapore; 2 Department of Chemical and Biomolecular Engineering, National University of Singapore, Singapore, Singapore; 3 IFReC, Osaka University, Osaka, Japan; 4 Temasek Life Sciences Laboratory, National University of Singapore, Singapore, Singapore; Università della Calabria, ITALY

## Abstract

Phytohormones play a critical role in mediating plant stress response. They employ a variety of proteins for coordinating such processes. In Arabidopsis thaliana, some members of a Cys-rich protein family known as C1-clan proteins were involved in stress response, but the actual function of the protein family is largely unknown. We studied At5g17960, a C1-clan protein member that possesses three unique C1 signature domains viz. C1_2, C1_3 and ZZ/PHD type. Additionally, we identified 72 other proteins in A. thaliana that contain all three unique signature domains. Subsequently, the 73 proteins were phylogenetically classified into IX subgroups. Promoter motif analysis of the 73 genes identified the presence of hormone-responsive and stress-responsive putative cis-regulatory elements. Furthermore, we observed that transcript levels of At5g17960 were induced in response to different hormones and stress treatments. At1g35610 and At3g13760, two other members of subgroup IV, also showed upregulation upon GA3, biotic and abiotic stress treatments. Moreover, seedlings of independent transgenic A. thaliana lines ectopically expressing or suppressing At5g17960 also showed differential regulation of several abiotic stress-responsive marker genes. Thus, our data suggest that C1-domain-containing proteins have a role to play in plant hormone-mediated stress responses, thereby assigning a putative function for the C1-clan protein family.

## Introduction

Being sessile organisms, plants are constantly exposed to various biotic and abiotic stress conditions. Plants demonstrate tolerance or resistance against such adverse conditions by the interplay of several fine-tuned strategies. One such strategy involves phytohormones that serve as key endogenous signals in mediating biotic and abiotic stress responses. The major hormones produced by plants are auxins, gibberellins (GA), cytokinins (Ck), abscisic acid (ABA), ethylene (ET), salicylic acid (SA) and jasmonates (JA). Synergistic and antagonistic interactions between different hormones, also known as hormone crosstalk, provide the plants with a powerful mechanism to regulate their defense response [1, 2]. For example, the negative regulators of GA signaling, *viz*., DELLA proteins (GAI, RGA, RGL1, RGL2 and RGL3) provide resistance to necrotrophs and susceptibility to virulent biotrophs by modulating the strength of SA and JA signaling [3]. Similarly, gain- and loss-of-function studies of ARABIDOPSIS HISTIDINE KINASEs (AHKs), the cytokinin receptors, showed that AHK1 acts as a positive regulator of drought and salinity response and also ABA signaling, whereas AHK2 and AHK3 negatively regulate osmotic stress response and ABA signaling [4].

The maintenance of such an intricate nexus of phytohormone signaling for plant defense response involves signal perception, signal transduction and eventually transcriptional regulation of target genes. Therefore, a better understanding of the stress response cascades necessitates a deeper insight into the gene regulatory networks. Fundamentally, transcriptional regulation requires binding of transcription factors (TFs) to their specific *cis*-regulatory elements present in the promoter region of target genes [5]. Thus, *cis*-elements are the functional DNA elements that control transcriptional activity. Analyses of upstream promoter regions of genes for the identification of *cis*-elements have been instrumental in understanding the major regulatory networks underlining different stress responses. For example, analysis of promoters of *A. thaliana* genes differentially expressed upon ABA or abiotic stress treatments identified ABA response elements [6]. Furthermore, c*is*-regulatory elements known to control osmotic stress response were used to identify other functionally relevant genes involved in the same process [7].

Members of specific gene families play key roles in mediating stress responses in plants. For example, *NtDC1A* and *NtDC1B*, two genes encoding divergent C1 (DC1)-domain-containing zinc-finger protein homologs, were identified as early response genes in tobacco BY-2 cells treated with glucan-enriched fraction from *Alternaria alternata* 102 [8]. Similarly, the wheat TaCHP, a Cys-, His- and Pro-rich zinc-finger protein with three C1 domains exhibited positive role in stress tolerance [9]. Furthermore, a single C1-domain-containing ULI3 protein was shown to be involved in UV-B signaling [10]. Although, these studies have established an association between C1-domain-containing proteins and stress response in plants, the general function of the protein family still remains unclear. Apparently, C1-domains share similarity to the N-terminal region of protein kinase C (PKC; a family of Ser/Thr protein kinases), but there is little evidence for the existence of PKC in plants [9]. In a previous microarray study conducted in our lab to determine genes specifically regulated by RGL2 (and hence GA) in *A. thaliana* [11], we identified a gene *At5g17960* that encodes a C1-domain protein whose role in stress responses may be studied further.

In this study, we show that At5g17960 possesses three C1 domains, *viz*., C1_2, C1_3 and ZZ/PHD type. We also identified 72 other proteins in *A. thaliana* that contain these three C1 domains and subsequently classified them into nine distinct subgroups based on their phylogenetic relationship. Promoter motif analyses of these 73 genes identified the presence of several hormone-responsive and stress-responsive putative *cis*-elements. Consequently, *At5g17960* was examined for its response to different plant hormones as well as various biotic and abiotic treatments. The gene showed significant upregulation in response to GA, SA, JA, ET and various stress conditions. We generated transgenic lines overexpressing and supressing *At5g17960* for studying the early molecular responses to various stress treatments. The transgenic seedlings exhibited differential expression profiles for selected stress-inducible marker genes. Thus, our data suggest that At5g17960 is a hormone-responsive C1-domain-containing protein that mediates abiotic and biotic stress responses in *A. thaliana*. Based on the data, we discuss the probable mode of action of C1-domain-containing proteins as a link between phytohormone signaling and plant stress responses.

## Materials and Methods

### Plant material and growth conditions

*A. thaliana* ecotype Columbia-0 (Col-0) (WT) plants were used in this study. The plants were grown at 22°C with 16 h light/8 h dark conditions at 75% RH. For seedling assays, the seeds were surface-sterilized by rinsing with 70% ethanol for 2 min followed by washing with 15% bleach for 5 min. Subsequently, the seeds were washed four times with sterile water. The surface sterilized, seeds were stratified at 4°C for 3 days in the dark. About 10–15 seeds were transferred to a 50 ml falcon tube with 10 ml Murashige and Skoog (MS) liquid medium (Caisson LABS) containing 1X MS and 2% glucose. The tubes containing the seeds were incubated at 22°C with shaking at 90 rpm under constant white light (~50 µE/m^2^/s) for growth.

### Plasmid construction

For *35S::At5g17960*, full-length coding sequence of *At5g17960* was PCR amplified using A15 FP and A15 RP and cloned into pJET vector (Thermo Scientific). Subsequently, the gene was released using XhoI/HindIII and cloned into pGREEN 35S vector digested with the same restriction enzymes. To construct amiR::*At5g17960*, amiRNA sequence (5’-GCGGGAAGCAAGTATCCACTT-3’) was designed using the amiRNA designer interface WMD [13, 14]. The sequence was introduced into Arabidopsis miR319a precursor by overlapping PCR using primers (A15_I_mIR, A15_II_mIR, A15_III_mIR, A15_IV_mIR) and pRS300 plasmid as the template. The resulting fragment was cloned into pJET vector and subsequently digested with XhoI/SpeI and ligated into XhoI/SpeI digested linearized pGREEN 35S vector. All constructs were verified by sequencing. Primers used are listed in the S1 Table.

### Transgenic plant generation

Transgenic *A. thaliana* plants were generated by *Agrobacterium tumefaciens* (strain GV3101:pMP90)-mediated floral dip method [12]. In the T1 generation, the selection of the plants was done by BASTA (2 ml/l) spray and the survivors were further tested for the presence of transgene by genotyping-PCR. The entire selection procedure was repeated in T2 generation for further verification of the transgenic lines selected in T1 generation. Subsequently, the T3 generation plants were used for expression analysis and transgenic lines with significantly high (overexpression construct) or low (suppression construct) transcript levels, compared to the wild-type, were selected for further analysis. For seedling assays, the seeds and seedlings were handled similar to the wild type.

### Phytohormone treatment

For GA_3_ treatment, 12-day-old WT seedlings were incubated in 1/2X liquid MS supplemented with 10 µM GA_3_ and samples were collected and snap frozen in liquid nitrogen after 30, 60 and 90 min of treatment. For ethylene treatment, 12-day-old seedlings were treated with 10 µM 1-aminocyclopropane-1-carboxylic acid (ACC) for 30 and 60 min. ACC stock was prepared in water. For SA and MeJA treatments also, seedlings were incubated in 1/2X MS liquid supplemented with 10 µM SA and 10 µM MeJA, respectively. However for SA treatment, samples were collected at 30, 60 and 180 min, whereas, MeJA, treatment was only given for 30 and 60 min as specified in the figure legends.

### Biotic and abiotic stress treatments

For biotic stress treatment, 12-day-old WT seedlings were treated with chitin at a final concentration of 10 mg/l in 1/2X MS liquid medium for 20, 30 and 60 min. Chitin solution was prepared by grinding shrimp shell chitin (Sigma Aldrich) to fine powder and subsequently, left overnight in sterile water for proper dissolution and finally filtered through muslin cloth.

Among the abiotic stress treatments, salt treatment was given by treating 12-day-old WT and transgenic seedlings with 200 mM NaCl or sterile water (mock treatment) in 1/2X MS solution for 20, 30 and 60 min. Drought treatment was given by leaving the seedlings in the laminar flow hood for 20 min to allow dehydration and subsequently transferred to 1/2X MS liquid medium for revival and samples were collected after 20 and 30 min time points. For UV irradiation treatment, seedlings were exposed to UV (30 W/cm^2^) for 10 min and then transferred back to liquid MS for revival and collected after 30 and 60 min time points. For cold treatment, 12-day-old seedlings were incubated at 10°C for 60 and 180 min.

### RNA extraction, cDNA synthesis and quantitative real time-PCR (qRT-PCR)

Total RNA was extracted from the treated seedlings using QIAGEN RNA extraction kit (QIAGEN) as per the manufacturer’s protocol. About 500 ng of RNA for each sample was reverse transcribed using Maxima First strand cDNA synthesis kit (Thermo Scientific) as per the manufacturer’s protocol. The cDNA samples were diluted 5-fold before use. The reaction mixture for qRT-PCR included 1 µl diluted cDNA, 5 µl 2x KAPA SYBR Master Mix (KAPA Biosystems), 0.2 µl each of the two primers (forward and reverse) and sterile water to a final volume of 10 µl. qRT-PCR was done using StepOne Real-Time PCR Systems (v2.1; Applied Biosystems) with denaturation at 95°C for 2 s followed by 40 cycles of denaturation at 95°C for 3 s and annealing and extension at 60°C for 30 s. The data analysis was done using StepOne software (v2.1; Applied Biosystems). For normalization of the data, amplification of *TUB2* gene was used as the internal control. Refer to Supplementary table (S1 Table) for information on primers used.

The expression analyses data represent means of three independent biological replicates each with three technical replicates. Statistical analysis was carried out using Student’s *t*-test (two-tailed) with level of significance P ≤ 0.05.

### General bioinformatic tools

Protein domain analysis was performed using Pfam 24.0 [15]. The genes encoding C1-domain-containing proteins were identified from the publically available PLAZA database using the Pfam ids [16]. Full-length amino acid sequence of all 73 proteins containing three C1 domains was extracted from TAIR (http://www.arabidopsis.org/) database. The ClustalW2 program was used for multiple sequence analysis of all the 73 proteins. Phylogenetic tree for the 73 sequences (amino acid) was constructed with MEGA v5.0 using the neighbor-joining (NJ) method and Poisson correction method [17, 18]. The protein weight matrix used was BLOSUM. All positions containing gaps and missing data were eliminated. Chromosome mapping was performed using the TAIR online Chromosome map tool.

### Identification of conserved Domains

Consensus sequences for the three conserved domains were verified by MEME version 4.9.0. [19]. For known protein domains, Eukaryotic Linear Motif (ELM) a functional site prediction tool was used [20]. The promoter sequences of the 73 genes covering the [-1000 to +200] regions relative to the experimentally validated transcription start site were extracted from our in-house *A. thaliana* promoter sequence database.

### Bioinformatic tools for motif detection

Promoter sequence motifs corresponding to previously identified or putative *cis*-elements were detected by the Dragon Motif Builder program with EM2 option [21]. Thirty motifs having length 8–10 nucleotides were detected with a threshold value of 0.875. Motifs were selected having more than 50% occurrence/presence at a threshold e value of ≤10^-3^ among the promoters investigated. The biological significance of these motifs was verified by their presence in Transcription Factor Binding databases such as TRANSFAC [22], PLACE [23] and AGRIS [24]. The percentage occurrences of all motifs belonging to the same TF family were added up to find out the total motif enrichment score.

## Results

### At5g17960 has C1_2, C1_3 and ZZ/PHD type C1 domains

Bioinformatic analyses of At5g17960 showed that it contains three of the five C1 signature domains proposed to be present in C1-clan (C1_1, C1_2, C1_3, C1_4 and ZZ) [15]. Pfam domain ids for each of the three C1 domains present in At5g17960 were retrieved from the TAIR database (S2 Table). Subsequently, using publicly available PLAZA database, we identified 72 other genes in *A. thaliana* genome that encode proteins containing all three unique C1 domains (C1_2, C1_3 and ZZ/PHD). We also scanned the PLAZA database for understanding the divergence of these domains in other plant species. Besides *A. thaliana*, *Oryza sativa* and *Populus trichocarpa* also showed significant divergence for the different C1 domains (S3 Table).

### Phylogenetic relationship between 73 C1-clan proteins in *A. thaliana*

**Figure 1:**
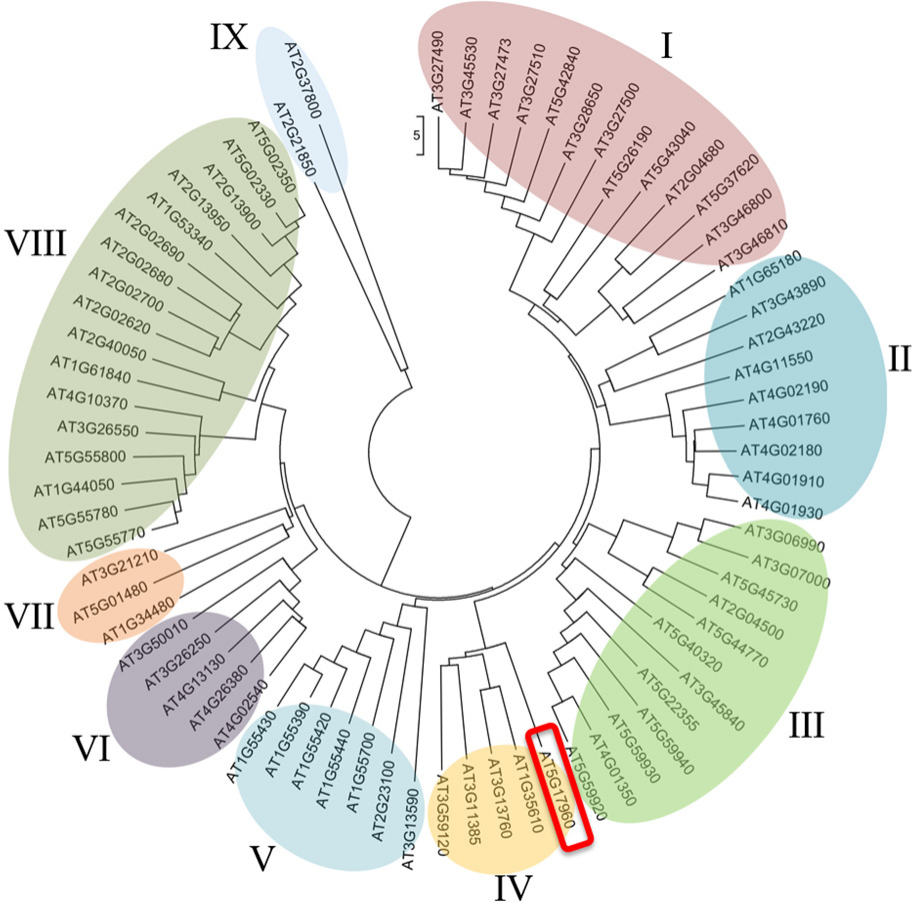
Unrooted circular phylogenetic tree of 73 genes of C1 clan. The tree was constructed using MEGA5 with the neighbor-joining method and poisson correction of three C1-clan domains namely C1_2, C1_3 and ZZ/PHD type in *Arabidopsis* [17, 18]. *At5g17960* is highlighted with a red box.

To study the phylogenetic relationship between 73 C1-clan proteins, a multiple sequence alignment of each of the three different C1 domains from all 73 proteins was done (S1 Fig.). An unrooted phylogenetic tree containing these 73 proteins was also generated (Fig. 1). Closely related proteins with high sequence similarity form a cluster based on common node are color-coded accordingly. Subsequently, we classified the 73 proteins of C1-clan into nine distinct subgroups based on common parental nodes shared by each subgroup. At5g17960, the protein of our interest, came under the subgroup IV along with four other proteins, namely, At1g35610, At3g13760, At3g11385 and At3g9120. Furthermore, we identified the consensus amino acid sequences of C1_2 domain (CX_2_CX_12_CX_2_CX_8_C: 29 amino acids), C1_3 domain (CX_2_CX_11_CX_2_CX_7_C: 27 amino acids) and ZZ/PHD type domain (CX_2_CX_11_CX_2_CX_7_CX_28_CX_2_C: 59 amino acids) (S2 Fig.).

To understand the evolution and divergence of the genes encoding these 73 C1-clan members, we mapped them on *A. thaliana* chromosomes. The genes were distributed in all five chromosomes of *A. thaliana*, but compared to other chromosomal arms, the density of distribution of the C1-clan genes was the least along the upper arm of Chromosome 1 (S3 Fig.). *At5g17960* is present on the short arm of chromosome 5.

### Promoter motif analysis of the 73 C1-clan genes in *A. thaliana*

To investigate the possible TFs involved in the transcriptional regulation of these 73 genes, we identified several conserved motifs present in the promoter regions (-1000 to +200 relative to TSS) of these genes. Among the motifs detected, the putative *cis*-elements such as GT element-like, GA response element (GARE)-like, Pyrimidine box-like, as-1/ocs-like, JA response element (JAre)-like, Auxin response element (AuxRE)-like, early response to dehydration stress (ERD1)-like, ARABIDOPSIS RESPONSE REGULATOR 10 (ARR10)-B element-like, W box-like, AAAG element-like and others were found to be highly enriched (Table 1). These putative *cis*-elements can be associated with TFs such as, MYB (GT1/GT3b), MYB (R1, R2R3), bZIP, ERF, ARF1, NAC, ARR-B, WRKY, DOF and others. The total enrichment scores for the different classes of TF are presented in Table 1. Percent occurrence among all 73 C1-clan genes, total information content of homology (TIC), E-value of homology with promoter database entry are provided in S4 Table.

**Table 1:** Detection of putative *cis*-elements and associated TFs in 73 C1-clan genes.

**Putative *cis*-elements detected**	**Associated TF**	Total motif enrichment score**
AT-hook/PE1 element-like	MYB (PF1)	533
GT-element-like	MYB (GT-1/GT-3b)	322
GARE-like	MYB (R1, R2R3)	206
Pyrimidine box-like	MYB (R1, R2R3)	136
Myb-box-like	MYB (R2R3)	119
TATA-element-like	TBP	306
as-1/Ocs/TGA-like	bZIP (Groups D, I, S)	260
JA response element-like	ERF (Gr. VI, VIII, IX)	245
AuxRE-like	ARF1	199
AAAG element-like	DOF	137
Sucrose Responsive Element (SURE)-like	WRKY (SUSIBA2)	131
ERD1-like	NAC	125
ARR10-B element-like	ARRB (ARR10)	79
W-box-like	WRKY (WRKY 18)	60
RNFG1 binding site-like	RNFG1	53
GAGA-like	GAGA-Binding factor	52

**Total motif enrichment score = sum of the % occurrences of all motif species belonging to the same TF family.

### Response of *At5g17960* to treatment with different phytohormones

**Figure 2:**
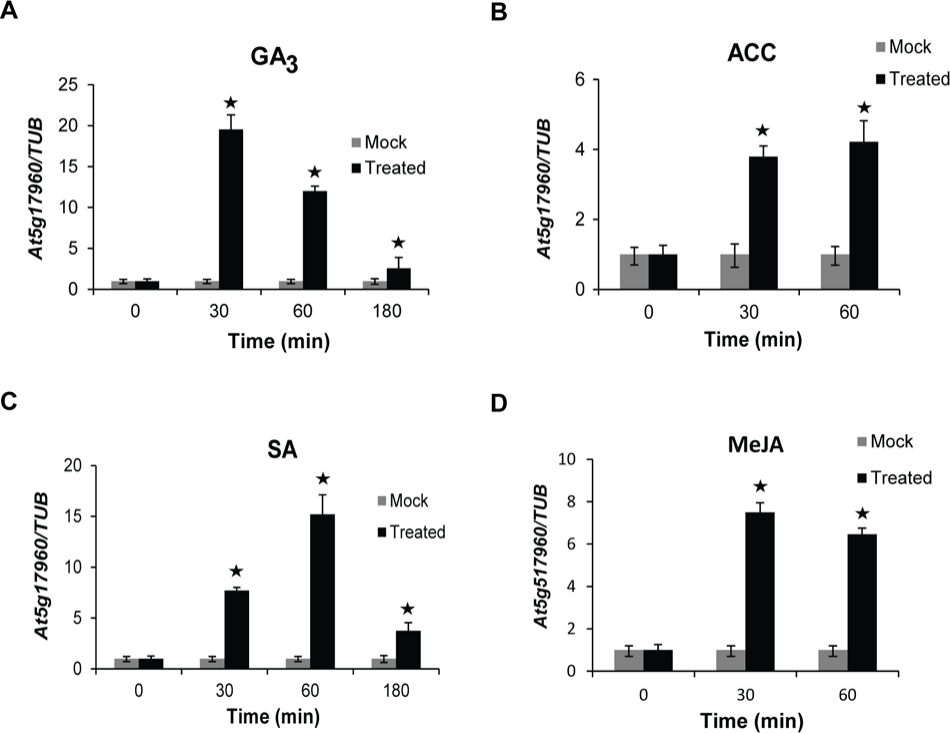
Response of *At5g17960* to different phytohormone treatments. The transcript levels of *At5g17960* were detected in 12-day-old WT seedlings at the indicated time points upon treatments with (A) 10 µM GA^3^, (B) 10 µM ACC, (C) 10 µM SA and (D) 10 µM MeJA. The mock treatments for GA3, SA and MeJA were done using ethanol, whereas, the mock treatment for ACC was done using sterile water. The expression levels were normalized against the expression of *TUB*. Data from three independent biological replicates were averaged and presented here with error bars representing SD. Bars with asterisks are significantly different from the mock treatments at the corresponding time points, as per Student’s *t*-test (P ≤ 0.05).

Promoter motif analyses identified significant enrichment of several hormone-responsive putative *cis*-elements, such as GARE-like (gibberellin response element), ARR10-B element (cytokinin response element) [25], JAre-like (JA response element) and as-1/ocs-like (SA response element) [26]. Hence, we were interested to investigate the regulation of *At5g17960* by different phytohormones. GA_3_ treatment to 12-day-old WT seedlings showed ~19-fold increase in the transcript levels of *At5g17960* after 30 min, in comparison to the mock treatment at the same time point (Fig. 2A). ACC treatment induced ~4-fold increase in the transcript levels of *At5g17960* after 30 min incubation which showed further increase after 60 min indicating *At5g17960* induction by ET (Fig. 2B). Upon SA treatment, the transcript levels increased ~7-fold after 30 min and ~15-fold after 60 min incubation, as compared to the mock treatments for the same time points (Fig. 2C). MeJA treatment also affected the expression levels of *At5g17960* with ~7-fold increase in the transcript levels after 30 min (Fig. 2D). These data clearly indicate that *At5g17960* is indeed induced by GA_3_, SA, JA and ET.

### Response of other members of subgroup IV to GA_3_ treatment

**Figure 3:**
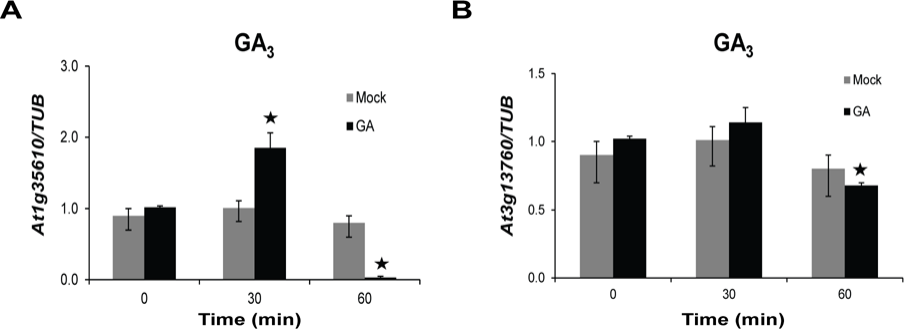
Response of *At1g35610* and *At3g13760* to GA^3^ treatment. The transcript levels of (A) *At1g35610* and (B) *At3g13760* were detected in 12-day-old WT seedlings at the indicated time points upon treatments with 10 µM GA^3^. The mock treatment was done using ethanol. The expression levels were normalized against *TUB* expression. Data presented here depict mean from three independent biological replicates with error bars representing SD. Bars with asterisks are significantly different from the mock treatments at the corresponding time points, as per Student’s *t*-test (P ≤ 0.05).

To further verify our promoter motif analysis data, we tested two other members of subgroup IV that are closest to *At5g17960* in the phylogenetic tree, *viz*., *At1g35610* and *At3g13760*, for their response to GA_3_ treatment (Fig. 1). GA_3_ was selected over other phytohormones used above, owing to the relatively high motif enrichment score of GARE-like elements compared to the putative *cis*-elements identified for other phytohormones (Table 1). In addition, the study was initiated with the identification of *At5g17960* from a GA-related microarray. Upon GA_3_ treatment, transcript levels of *At1g35610* in 12-day-old WT seedlings depicted ~2-fold upregulation at 30 min time point, as compared to the mock treatment (Fig. 3A). However, the gene showed significant downregulation at 60 min time point, compared to the mock treatment. *At3g13760* transcript levels, on the other hand, did not show any difference after 30 min, but showed a minor, yet significant downregulation at 60 min time point, upon GA_3_ treatment (Fig. 3B).

### Response of *At5g17960* to different biotic and abiotic stresses

**Figure 4:**
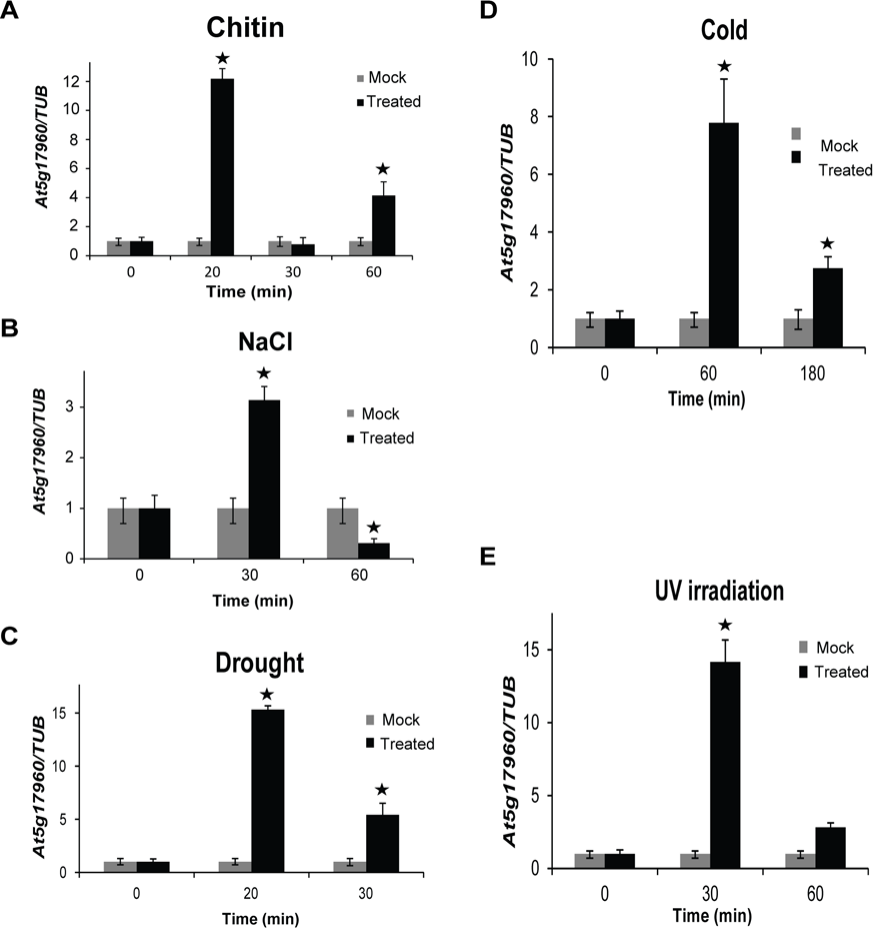
Response of *At5g17960* to different stress treatments. The expression levels of *At5g17960* were examined in 12-day-old WT seedlings at the indicated time points upon exposure to biotic (chitin) and different abiotic (salinity, drought, cold and UV) treatments. (A) 10 mg/L chitin solution was used for biotic stress. The mock treatment was done using sterile water. (B) For salinity treatment, 200 mM NaCl was used and mock treatment was done using sterile water. (C) Drought treatment was given by desiccating the seedlings for 20 min and then allowing them to recover for the indicated time points. (D) Seedlings were kept at 10°C for exposing them to cold treatment. (E) For UV treatment, seedlings were exposed to 30 W/cm^2^ UV radiation for 10 min and then allowed to recover for indicated time points. Mock for drought, cold and UV treatments represent untreated seedlings. The expression levels were normalized against the expression of *TUB*. Data from three independent biological replicates were averaged and presented here with error bars representing SD. Bars with asterisks are significantly different from the mock treatments at the corresponding time points, as per Student’s *t*-test (P ≤ 0.05).

Since promoter motif analyses of the 73 C1-clan genes also identified several stress-responsive putative *cis*-elements, we examined the response of *At5g17960* upon biotic and abiotic stress treatments. Chitin treatment of 12-day-old WT seedlings showed significant increase in *At5g17960* transcript level within 20 min (Fig. 4A), suggesting its possible role in early signaling.

Upon salt treatment with 200 mM NaCl, a ~3-fold increase in the transcript levels of *At5g17960* was observed at 30 min time point, as compared to the mock treatment at 30 min (Fig. 4B). Similarly, a ~15-fold increase in the transcript levels of *At5g17960* was observed when the seedlings were exposed to drought conditions for 20 min and then allowed to recover in MS solution for 20 min (Fig. 4C). However, the transcript levels showed only ~5-fold increase when the revival time was increased to 30 min. Cold treatment induced an increase of ~8-fold and ~3-fold in the transcript levels of *At5g17960* at 60 min and 180 min, respectively (Fig. 4D). A ~14-fold increase in the transcript levels was observed upon UV irradiation for 10 min followed by 30 min recovery time in liquid MS (Fig. 4E). Taken together, the data strongly suggest that *At5g17960* is rapidly and significantly induced by various biotic and abiotic stresses.

### Response of *At1g35610* and *At3g13760* to biotic and abiotic stress conditions

**Figure 5:**
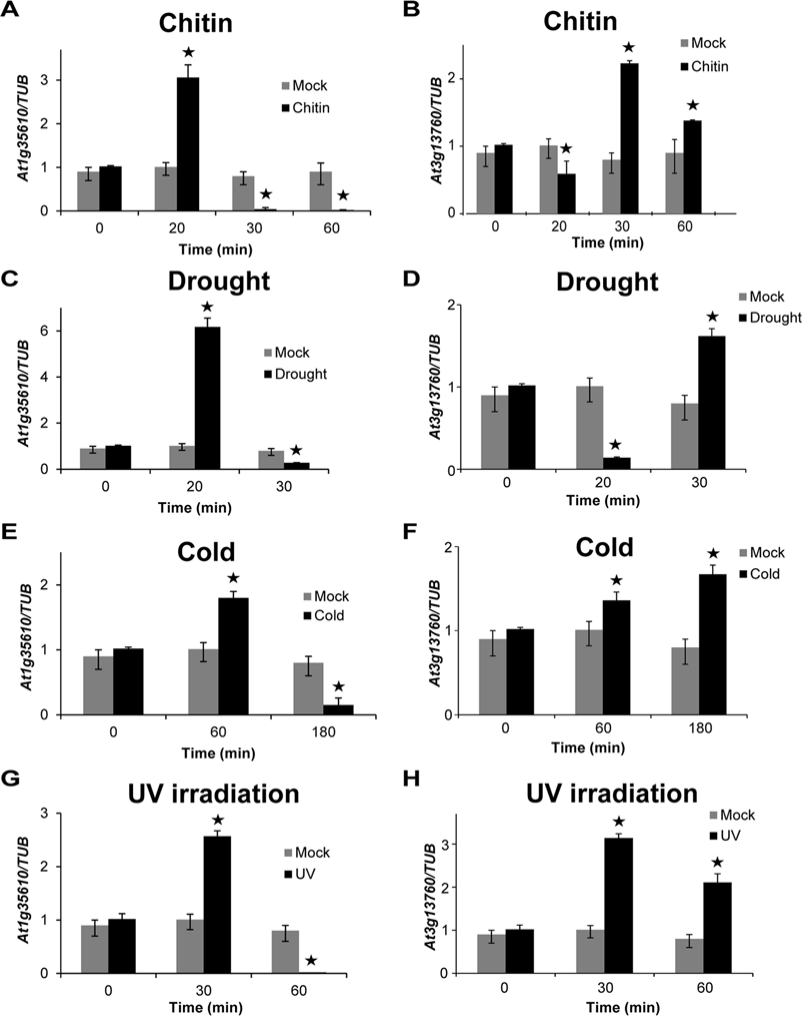
Response of *At1g35610* and *At3g13760* to different stress treatments. The expression levels of *At1g17960* and *At3g13760* were examined in 12-day-old WT seedlings at the indicated time points upon exposure to biotic (chitin) and different abiotic (drought, cold and UV) treatments. (A) Transcript levels of *At1g17960* and (B) *At3g13760* were examined at indicated time points upon treatment with 10 mg/L chitin solution. The mock treatment was done using sterile water. (C) Transcript levels of *At1g17960* and (D) *At3g13760* were checked after exposing the WT seedlings to 20 min desiccation and then allowing them to recover for the indicated time points. (E) Transcript levels of *At1g17960* and (F) *At3g13760* were examined in WT seedlings exposed to cold treatment (10°C) for indicated time points. (G) Transcript levels of *At1g17960* and (H) *At3g13760* in seedlings exposed to 30 W/cm^2^ UV radiation for 10 min were examined at different time points. Mock for drought, cold and UV treatments represent untreated seedlings. The expression levels were normalized against the expression of *TUB*. Data from three independent biological replicates were averaged and presented here with error bars representing SD. Bars with asterisks are significantly different from the mock treatments at the corresponding time points, as per Student’s *t*-test (P ≤ 0.05).

To verify if two other members of subgroup IV also respond to stress conditions, we examined the induction of *At1g35610* and *At3g13760* upon chitin treatment to WT seedlings. *At1g35610* exhibited ~3-fold upregulation at 20 min time point, whereas, *At3g13760* showed ~2-fold upregulation at 30 min (Fig. 5A and B).

Among abiotic stress conditions, drought treatment caused ~6-fold upregulation of *At1g35610* after 20 min and ~1.5-fold upregulation of *At3g13760* after 30 min of recovery time (Fig. 5C and D). Upon cold treatment, a ~1.8-fold increase of *At1g35610* transcripts and ~1.5-fold increase of *At3g13760* transcripts was noticed at 60 min time point (Fig. 5E and F). Similarly, a 10 min UV exposure followed by 30 min recovery in MS solution caused an increase of ~2.5-fold in the transcript levels of *At1g35610* and ~3-fold increase in the transcript levels of *At3g13760* (Fig. 5G and H). Altogether, the data strongly suggest that both genes, *At1g35610* and *At3g13760*, show significant alteration in regulation upon biotic (chitin) and abiotic (drought, cold and UV) stress treatments within 60 min.

### Response of overexpression and suppression of *At5g17960* on stress-responsive marker genes

**Figure 6:**
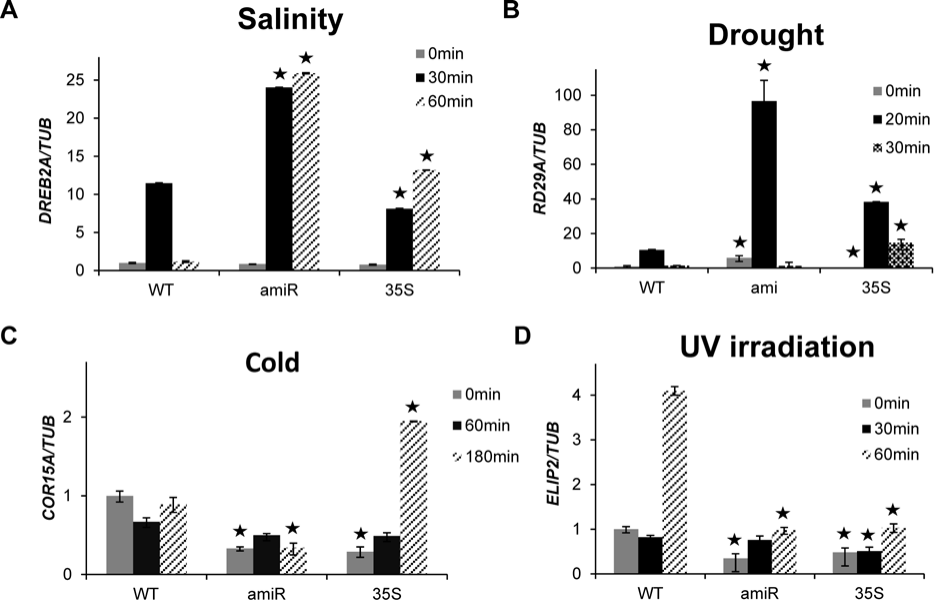
Response of stress-inducible marker genes in transgenic *At5g17960* seedlings upon abiotic stress treatments. (A) Expression levels of *DREB2A* in 12-day-old seedlings of the WT, amiR::*At5g17960* #1–1 and 35S::*At5g17960* #9–1 upon treatment with 200 mM NaCl were examined at different time points. (B) Expression levels of *RD29A* were checked upon exposure of the seedlings to 20 min drought treatment. (C) *COR15A* expression levels were examined upon exposure of the WT, amiR::*At5g17960* #1–1 and 35S::*At5g17960* #9–1 seedlings to 10°C for indicated time points. (D) Expression levels of *ELIP2* in the WT and transgenic seedlings were examined after exposing the seedlings to 10 min UV irradiation. The expression levels were normalized against the expression of *TUB*. Data from three independent biological replicates were averaged and presented here with error bars representing SD. Bars with asterisks are significantly different from the WT at the corresponding time points, as per Student’s *t*-test (P ≤ 0.05).

To examine if altered expression of *At5g17960* affects stress response, we generated transgenic lines with 35S::*At5g17960* (for ectopically expressing *At5g17960*) and amiR::*At5g17960* (for suppressing *At5g17960*). For each construct at least 7 independent transgenic lines were obtained (S4 Fig.). The transgenic lines did not show any significant phenotypic changes from the wild type under normal growth conditions. Since we were focusing on the early molecular responses of these lines to different stress treatments, 12-day-old seedlings of the 35S line (35S::*At5g17960* #9–1) and amiR line (amiR::*At5g17960* #1–1) were used for functional analyses. Furthermore, to study the impact of overexpression and suppression of *At5g17960* on stress response, we selected well-characterized stress-responsive marker genes, one for each of the four abiotic stress conditions, *DREB2A* (salt stress), *RD29A* (drought stress), *COR15A* (cold stress) and *ELIP2* (UV stress) [27, 28]. Upon salt stress with 200 mM NaCl, a strong induction of *DREB2A* was observed in the amiR line at both 30 and 60 min time points, as compared to the WT (Fig. 6A). However, in the 35S seedlings, the differences in the transcript levels of *DREB2A* were of a lower magnitude than the amiR line. When the 35S and amiR seedlings were subjected to drought treatment, we noticed ~9-fold increase in the expression levels of *RD29A* in the amiR line at 20 min time point, compared to its expression in the WT at 20 min (Fig. 6B). On the other hand, ectopic expression line showed only ~3.5-fold enhancement in the transcript levels of *RD29A* at 20 min time point, compared to its levels in the WT at 20 min. Upon cold treatment, amiR seedlings showed only minor differences in the transcript levels of *COR15A* (Fig. 6C). However, 35S seedlings showed a ~2-fold induction of *COR15A* at 180 min, compared to its levels in the WT at the corresponding time point. UV irradiation treatment to 35S and amiR seedlings showed only minor changes in the expression levels of *ELIP2* at 30 min time point, compared to the WT. However, both transgenic lines showed significant downregulation of *ELIP2* at 60 min time point, in comparison to its levels in the WT (Fig. 6D). The aforementioned results clearly highlight that modulating the transcript levels of *At5g17960* affects the expression levels of different stress-responsive marker genes and hence, this may play a key role in mediating stress response in plants.

## Discussion

Abiotic and biotic stress conditions not only affect plant growth and development adversely, but also affect huge yield losses worldwide [29]. To counteract such stress conditions, plants have naturally evolved adaptive response mechanisms and strategies that allow them to survive. Nevertheless, these adaptations to adverse conditions do not adequately benefit the plants to overcome recurring crop yield losses owing to the intensive agricultural practices currently in place. This necessitates a better understanding of the molecular mechanisms underlining the plant stress response which can be used as control-switches for improving stress tolerance as well as crop yield. Several studies have established, beyond doubt, the significance of hormonal crosstalk in mediating plant stress response [1]. However, the intricacies of the involvement of such signaling networks demand comprehensive research in the field [30]. To understand such intrinsic behaviour, our analyses provide significant information on the potential involvement of C1-clan gene family containing a number of putative *cis*-elements (including hormonal- and stress-responsive elements) in hormonal crosstalk-mediated plant stress response.

Multiple sequence alignment analysis, phylogenetic classification and conserved motif analysis of the 73 proteins possessing C1 domains were done in accordance with an earlier study where similar strategies were employed for AP2/ERF TFs which helped to predict their function based on sequence similarities (Figs. 1, S1 and S2) [31]. The phylogenetic classification was supported by similar trends in expression patterns of *At5g17960*, *At1g35610* and *At3g13760* in response to different stress treatments, because proteins encoded by the three genes are clustered together in the same subgroup IV of the phylogenetic tree (Figs. 1, 4 and 5). However, response of the three genes to GA_3_ treatment was not alike, because *At3g13760* did not get induced by GA_3_ (Fig. 3B), supporting the view that individual members of the gene family show differential hormone response. However, the experimental observations together with our promoter analysis results on the presence of multiple putative *cis*-elements for different phytohormones, provides further credence to the potential role of C1-clan members in coordinating hormonal crosstalk to mediate diverse stress responses.

Exploiting the presence of putative *cis*-regulatory elements known to be involved in a particular process for assigning putative functions to uncharacterized genes has been used for identifying functionally relevant genes [6, 7]. In our analysis, presence of hormone-responsive and stress-responsive *cis*-regulatory elements in the promoter regions of 73 C1 clan genes has provided significant information to elucidate a potential role of C1-domain-containing proteins in our study (Table 1). The suggested involvement of C1 clan genes in hormone response was validated by upregulation of *At5g17960* in response to GA_3_, SA, JA and ET and also induction of *At1g35610* upon GA_3_ treatment (Figs. 2 and 3A). Additionally, the participation of C1 clan genes in stress response was confirmed by upregulation of *At5g17960*, *At1g35610* and *At3g13760* in response to biotic and abiotic stress treatments (Figs. 4 and 5). In addition, identification of highly enriched putative *cis*-elements and their cognate TFs also significantly supported the experimental observations, as NAC, MYB and WRKY are well-characterized stress-responsive TFs (Table 1) [32]. Our claim was further validated by the differential regulation of stress-inducible marker genes (*DREB2A*, *RD29A*, *COR15A* and *ELIP2*) in 35S::*At5g17960* #9–1 and amiR::*At5g17960* #1–1 transgenic seedlings, upon stress treatments (Fig. 6). The aforementioned data are evidently in agreement with the few existing reports about the involvement of C1-domain proteins in stress response [9, 28].

In conclusion, the poorly studied C1-clan protein family appears to play key roles in the hormone-mediated stress response in *A. thaliana*. The presence of multiple putative *cis*-elements, response of the family members to different phytohormones and stress treatments and expression levels of stress-responsive marker genes in transgenic plants with altered *At5g17960* expression have provided important information for elucidating the potential involvement of the gene family in hormone-mediated stress remediation. In addition, our study delivers significant information for further in depth functional analyses of the stress-responsive C1-clan gene family. Further studies are needed to understand how the mature plants will respond to various stresses. Such studies have the potential to open avenues for the development of novel crop improvement strategies.

## Supporting Information

S1 Table List of primers used in the study. (PDF)

S2 Table Pfam and InterPro Ids of five different C1-clan domains. (PDF)

S3 Table Summary of C1-clan members present in different plant species. (PDF)

S4 Table Detection of putative *cis*-elements in the promoters of 73 C1-clan genes. (PDF)

S1 Fig Multiple sequence alignment of three C1 domains of 73 C1-clan proteins. The multiple sequence alignment of (A) C1_2 domain, (B) C1_3 domain and (C) ZZ/PHD domain 73 C1-clan proteins of *Arabidopsis thaliana*. The gene name is followed by amino acid position, followed by P-value and the amino acids corresponding to C1_2 domain are highlighted in color.
(PDF)

S2 Fig Consensus sequence of C1_2, C1_3 and ZZ/PHD type domains. (A) The C1_2 consensus sequence is from amino acid 1–29, in total 29aa. (B) The C1_3 the consensus sequence is from amino acid 26–52, in total 27aa. (C) The ZZ/PHD type the consensus sequence is from amino acid 2–60, in total 59aa. The derived consensus sequence is shown below each diagram. X-axis refers to position of amino acid. Y-axis depicts the degree of consensus based on the size of amino acid i.e. the larger the size of amino acid symbol the more conserved it is.
(PDF)

S3 Fig Location of 73 C1-clan genes on *A. thaliana* chromosomes. The 73 genes were mapped to the five chromosomes of *A. thaliana*. The green bars represent the *A. thaliana* chromosomes (numbered one to five at the top of each bar). The 73 genes are represented by their accession numbers. Chromosome mapping was performed using the TAIR online Chromosome map tool.
(PDF)

S4 Fig Expression of *At5g17960* transcripts in transgenic plants. (A) Transcript levels of *At5g17960* were significantly upregulated in the leaves of 7 selected *35S-At5g17960* independent lines in the T3 generation. (B) Transcript levels of *At5g17960* were significantly decreased in the leaves of 8 selected *amiRNA-At5g17960* independent lines in the T3 generation. Transcript levels in (A) and (B) were determined by qRT-PCR and are shown relative to *TUB2* expression. Values are the mean ± standard deviation of three independent biological replicates each with three technical replicates.
(PDF)
