# Supplementary material for: A Hormone-Responsive C1-Domain-Containing Protein At5g17960 Mediates Stress Response in Arabidopsis thaliana
Source: PLoS One. 2015 Jan 15;10(1):e0115418. doi: 10.1371/journal.pone.0115418 (PMC4295845; doi:10.1371/journal.pone.0115418)
Supplement: S1 Table — (PDF) [file pone.0115418.s001.pdf]

Supporting Information (Ravindran Vijay Bhaskar et al.)

Table S1. List of primers used in the study.

Gene Name	Primer name	Primer Sequence (5'-3')
At5g17960	A15 FP	CTCGAGATGGAAGAGCCTAAGAATATA
	A15 RP	AAGCTTCTATAAACATTTTAATAAAAGACA
	A15 INT FP1	TGCACACAAACCAAACCCCC
	A 15 INT RP1	GGACTTTGTCAATGCTATAC
	A15 RT FP	TCGTTTGGCTCATCCGACTCATCCT
	A15 RT RP	TCCACATCGCAGATGGTGCAGTG
	A15_I_mIR	GAGCGCGAAGCAAGTATCCACTATCTCTCTTTTGTATTCC
	A15_II_mIR	GATAGTGGATACTTGTTCGCGCTCAAAGAGAATCAATGA
	A15_III_mIR	GATAATGGATACTTGGTTCGCGCTCACAGCTCGTGATATG
	A15_IV_mIR	GAGCGCGAACCAAGTATGCATTATCTACATATATATTCCT
At1g35610	3A RT FP	ACCGGTTTGATCTTCTACGAAGGCT
	3A RT RP	TCGGAAGACGGATGAGACGACG
At3g13760	3B RT FP	ACGGTGGAAGTTTTCCGCAACAG
	3B RT RP	GCCATCTCATGCTGCTCGTAGCC
PR3	PR3 RT FP	TGGTGTTAATCCTGGTGGTAATCT
	PR3 RT RP	ACTAAATAGCAGCTTCGAGGAGG
PDF1.2a	PDF1.2a RT FP	TGTAACAACAACGGGAAAATAAACA
	PDF1.2a RT RP	TTTGCTTCCATCATCACCTTTATCT
COR15A	COR15A RT FP	CTCAGTTCGTCGTCGTTTC
	COR15A RT RP	CATCTGCTAATGCCTCTTT
RD29A	RD29A RT FP	CCAATAAACGTGGACCGACT
	RD29A RT RP	CTCTCTACGTGGCTATGCGA
ELIP2	ELIP2 RT FP	CCACAGTCTCCTCCTCCA
	ELIP2 RT RP	TGCTAGTCTCCCGTTGATC
DREB2A	DREB2A RT FP	TGAAAGGTAAAGGAGGAC
	DREB2A RT RP	CTTCTTGAGCAGTAGGGA
