# Supplementary material for: A Hormone-Responsive C1-Domain-Containing Protein At5g17960 Mediates Stress Response in Arabidopsis thaliana
Source: PLoS One. 2015 Jan 15;10(1):e0115418. doi: 10.1371/journal.pone.0115418 (PMC4295845; doi:10.1371/journal.pone.0115418)
Supplement: S2 Table — (PDF) [file pone.0115418.s002.pdf]

Supporting Information (Ravindran Vijay Bhaskar et al.)

Table S2. Pfam and InterPro Ids of five different C1-clan domains.

	C1_1	C1_2/DC1	C1_3/C1-like	C1_4	ZZ/PHD type
**Pfam Id**	PF00130	PF03107	PF07649	PF07975	PF00569
**InterPro Id**	IPR002219	IPR004146	IPR011424	IPR004595	IPR001965
