# Supplementary material for: A Hormone-Responsive C1-Domain-Containing Protein At5g17960 Mediates Stress Response in Arabidopsis thaliana
Source: PLoS One. 2015 Jan 15;10(1):e0115418. doi: 10.1371/journal.pone.0115418 (PMC4295845; doi:10.1371/journal.pone.0115418)
Supplement: S3 Table — (PDF) [file pone.0115418.s003.pdf]

Supporting Information (Ravindran Vijay Bhaskar et al.)

Table S3. Summary of C1-clan members present in different plant species.

	C1_1	C1_2	C1_3	C1_4	ZZ type
Arabidposis thaliana	63	136	145	1	163
Oryza sativa japonica	13	2	14	1	84
Medicago trancatula	3	4	7	1	71
Vitis vinifera	5	1	5	1	71
Zea mays	6	-	7	1	107
Volvox carteri	1	-	-	1	22
Brachypodium distachyon	3	1	-	1	82
Populus trichocarpa	22	24	33	1	116
Sorghum bicolor	7	2	7	1	93

"-" indicates no gene identified with the respective domain in the genome
