# Supplementary material for: A Hormone-Responsive C1-Domain-Containing Protein At5g17960 Mediates Stress Response in Arabidopsis thaliana
Source: PLoS One. 2015 Jan 15;10(1):e0115418. doi: 10.1371/journal.pone.0115418 (PMC4295845; doi:10.1371/journal.pone.0115418)
Supplement: S4 Table — (PDF) [file pone.0115418.s004.pdf]

Supporting Information (Ravindran Vijay Bhaskar et al.)

Table S4. Detection of putative *cis*-elements in the promoters of 73 C1-clan genes.

Motifs	cis-elements	Associated TF	% (TIC), E-value*	Total motif enrichment score**
AT-hook/PE1 element-like	AAAAATTA<br>TTGTTTTTTT<br>TTTTTTCCA<br>TAGTTTTT<br>CTTTTTTTTT<br>AAAAATTA<br>TTTTTTGA<br>AAAAATATTA	MYB (PF1)<br>MYB (PF1)<br>MYB (PF1)<br>MYB (PF1)<br>MYB (PF1)<br>MYB (PF1)<br>MYB (PF1)<br>MYB (PF1)	81 (13.27), 1e-003<br>73 (14.87), 1e-004<br>70 (13.52), 9e-004<br>67 (13.23), 3e-004<br>66 (17.49), 1e-004<br>60 (13.62), 2e-005<br>60 (15.06), 3e-004<br>56 (15.23), 3e-004	533
GT-element-like	TTTGTTTTA<br>TAGTTTTT<br>CTTTTTTTTT<br>ATGTGAAA<br>AAACCATG	MYB (GT-3b)<br>MYB (GT-1)<br>MYB (GT-1/GT-3b)<br>MYB (GT-1)<br>MYB (GT-1)	71 (13.45), 3e-003<br>67 (13.23), 3e-004<br>66 (17.49), 1e-004<br>66 (13.11), 3e-005<br>52 (12.91), 2e-004	322
GARE-like	TTGTTTTTTT<br>TTTGTTTTA<br>AACAATTA	MYB (R1, R2R3)<br>MYB (R1, R2R3)<br>MYB (R1, R2R3)	73 (14.87), 1e-004<br>71 (13.45), 3e-003<br>62 (13.00), 2e-004	206
Pyrimidine box-like	TTTTTTCCA<br>CTTTTTTTTT	MYB (R1, R2R3)<br>MYB (R1, R2R3)	70 (13.52), 9e-004<br>66 (17.49), 1e-004	136
Myb-box-like	AAACCATG<br>TAGTTTTT	MYB (R2R3)<br>MYB (R2R3)	52 (12.91), 2e-004<br>67 (13.23), 3e-004	119
TATA-element-like	ATAAATAT<br>TATAATAA<br>AACATTAAA<br>AAAAATATTA<br>GTATAATA	TBP<br>TBP<br>TBP<br>TBP<br>TBP	70 (13.72), 2e-004<br>66 (14.04), 4e-004<br>59 (13.97), 6e-005<br>56 (15.23), 3e-004<br>55 (12.86), 6e-005	306
As1/Ocs/TGA-like	ACATGATTA<br>ATGTGAAA<br>TTTTTTGA<br>TATATGAA	bZIP (Groups D, I, S)<br>bZIP (Groups D, I, S)<br>bZIP (Groups D, I, S)<br>bZIP (Groups D, I, S)	79 (12.37), 4e-004<br>66 (13.11), 3e-005<br>60 (13.62), 2e-005<br>55 (13.83), 2e-004	260
JA response element-like	ACATGATTA<br>TTTTTTGA<br>TATATGAA<br>TCAAAATTT	ERF (Gr. VI, VIII, IX)<br>ERF (Gr. VI, VIII, IX)<br>ERF (Gr. VI, VIII, IX)<br>ERF (Gr. VI, VIII, IX)	79 (12.37), 4e-004<br>60 (13.62), 2e-005<br>55 (13.83), 2e-004<br>51 (14.07), 3e-004	245
AuxRE-like	TTGTTTTTTT<br>TTTGTTTTA<br>TATATGAA	ARF1<br>ARF1<br>ARF1	73 (14.87), 1e-004<br>71 (13.45), 3e-003<br>55 (13.83), 2e-004	199

AAAG element-like	TCTTTCACC<br>CTTTTTTTTT	DOF1, DOF2<br>DOF1/DOF4/DOF11/DOF22	71 (12.17), 3e-004<br>66 (17.49), 1e-004	137
Sucrose Responsive Element (SURE)-like	GAAGAAAA<br>AAAAATTG	WRKY (SUSIBA2)<br>WRKY (SUSIBA2)	74 (13.66), 2e-004<br>59 (13.32), 9e-005	131
ERD1-like	ATGTGAAA<br>AAAAATTG	NAC<br>NAC	66 (13.11), 3e-005<br>59 (13.32), 9e-005	125
ARR10-binding element-like	ACATGATTA	ARRB (ARR10)	79 (12.37), 4e-004	79
W-box-like	TTTTTTGA	WRKY (WRKY 18)	60 (13.62), 2e-005	60
RNFG1 binding site-like	TCCATCGA	RNFG1	53 (11.08), 8e-005	53
GAGA-like	TTCTCATA	GAGA-Binding factor	52 (13.34), 6e-004	52

*% = percent occurrence among all 73 C1 clan genes, TIC = total information content of homology, E-value = E-value of homology with promoter database entry
**Total motif enrichment score = sum of the % occurrences of all motif species belonging to the same TF family
