# Supplementary material for: A Hormone-Responsive C1-Domain-Containing Protein At5g17960 Mediates Stress Response in Arabidopsis thaliana
Source: PLoS One. 2015 Jan 15;10(1):e0115418. doi: 10.1371/journal.pone.0115418 (PMC4295845; doi:10.1371/journal.pone.0115418)
Supplement: S1 Fig — The multiple sequence alignment of (A) C1_2 domain, (B) C1_3 domain and (C) ZZ/PHD domain 73 C1-clan proteins of Arabidopsis thaliana. The gene name is followed by amino acid position, followed by P-value and the amino acids corresponding to C1_2 domain are highlighted in color. (PDF) [file pone.0115418.s005.pdf]

Supporting Information (Ravindran Vijay Bhaskar et al.)

Figure S1

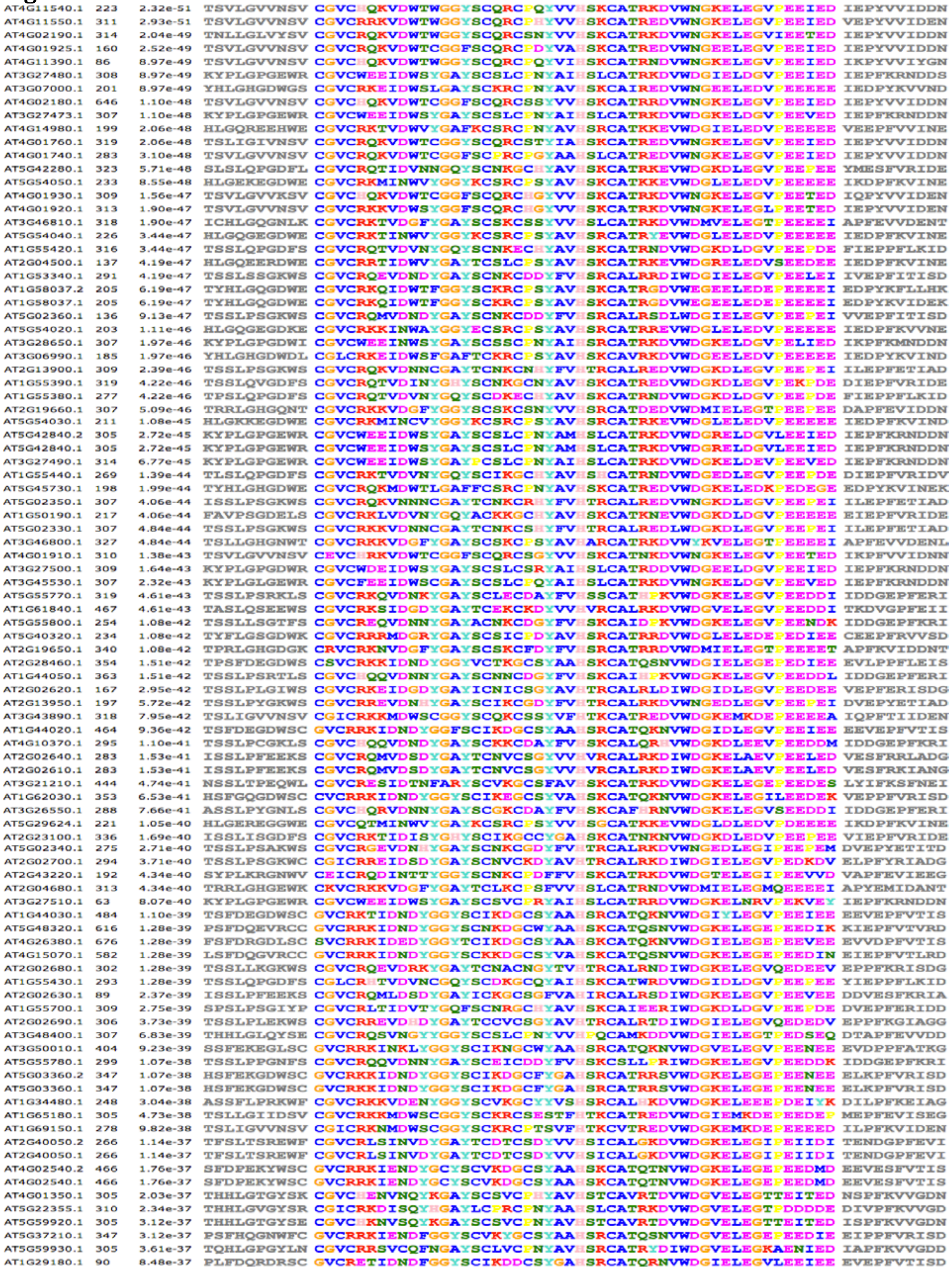

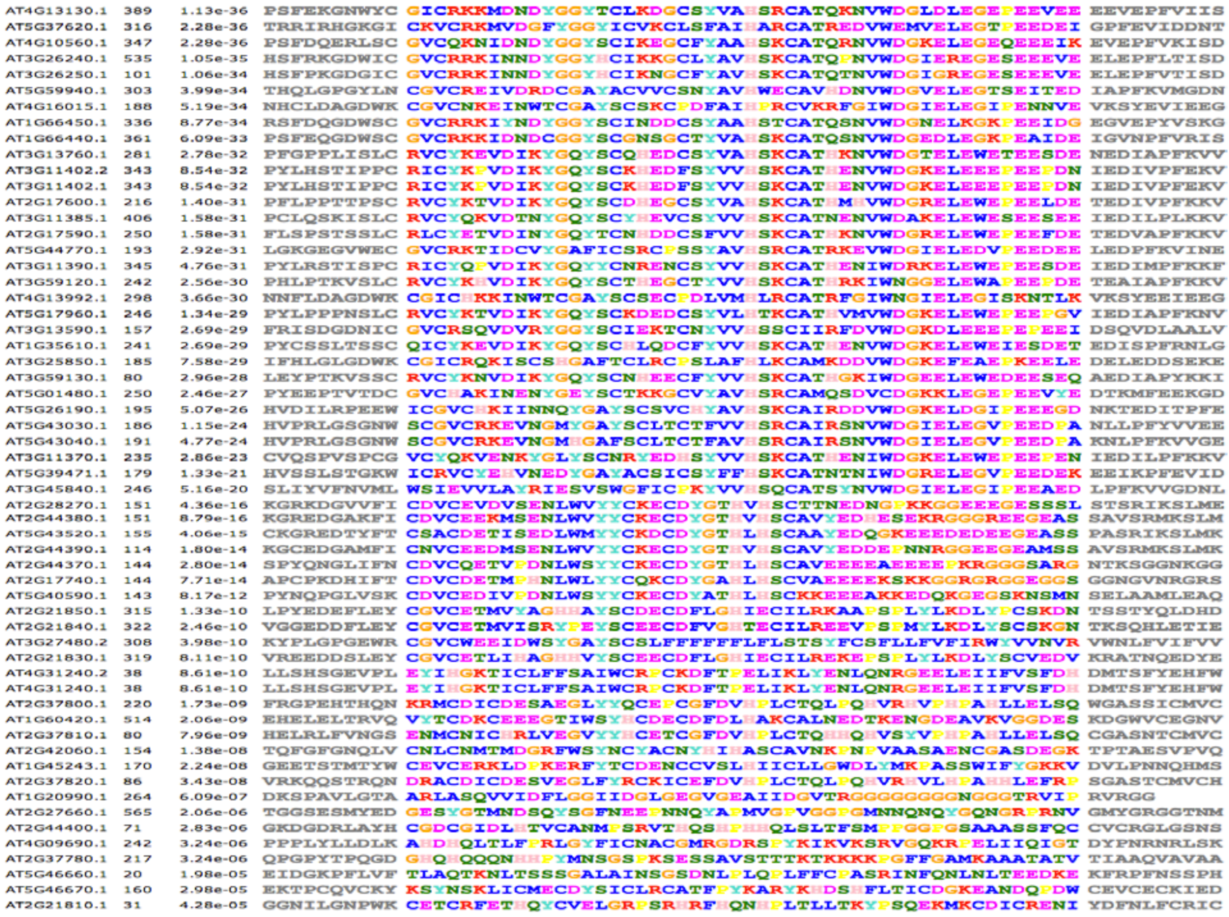

Figure S1A. Multiple sequence alignment of C1_2 domain of 73 C1-clan genes.
The multiple sequence alignment of C1_2 domain of 73 C1 clan genes in *Arabidopsis thaliana*. The gene name is followed by amino acid position, followed by P-value and the amino acids corresponding to C1_2 domain are highlighted in color.

AT4G11550.1	311	4.53e-52	TSVLGVVNSV	CGVCRRKVDWTWGGYSCQRCPHYVVHSKCATREDVWNGKELEGVPEEIED	VEPYVVIDEN
AT4G11540.1	223	7.31e-52	TSVLGVVNSV	CGVCHQKVDWTWGGYSCQRCPQYVVHSKCATRKDVWNGKELEGVPEEIED	IEPYVVIDDN
AT4G01925.1	160	8.38e-50	TSVLGVVNSV	CGVCRQKVDWTCGGFSCQRCPDYVAHSKCATREDVWNGEELEGVPEEIED	IEPYVVIDDN
AT4G02190.1	314	1.04e-49	TNLLGLVYSV	CGVCRQKVDWTWGGYSCQRCSNYVVHSKCATRKDVWNGKELEGVIEETED	IEPYVVIDDN
AT4G11390.1	86	3.00e-49	TSVLGVVNSV	CGVCHQKVDWTWGGYSCQRCPQYVIHSKCATRNDVWNGKELEGVPEEIED	IKPYVVIYGN
AT4G02180.1	646	3.69e-49	TSVLGVVNSV	CGVCHQKVDWTCGGFSCQRCSSYVVHSKCATRRDVWNGKELEGVPEEIED	IEPYVVIDDN
AT3G07000.1	201	8.46e-49	YHLGHGDWGS	CGVCRKEIDWSLGAYSCKRCPNYAVHSKCAIREDVWNGEELEDVPEEEEE	IEDPYKVVND
AT3G27480.1	308	1.04e-48	KYPLGPGEWR	CGVCWEEIDWSYGAYSCSLCPNYAIHSLCATRKDVWDGIELDGVPEEIED	IEPFKRNDDS
AT3G27473.1	307	1.28e-48	KYPLGPGEWR	CGVCWEEIDWSYGAYSCSLCPNYAIHSLCATRKDVWDGKELDGVPEEVED	IEPFKRNDDN
AT4G01760.1	319	1.56e-48	TSLIGIVNSV	CGVCRQKVDWTCGGYSCQRCSTYIAHSKCATREDVWNGKELEGVPEEIED	IEPYVVIDDN
AT5G42280.1	323	1.92e-48	SLSLQPGDFL	CGVCRQTIDVNNGQYSCNKGCHYAVHSKCATRKEVWDGKDLEGVPEEPEE	YMESFVRIDE
AT4G01740.1	283	4.28e-48	TSVLGVVNSV	CGVCRQKVDWTCGGFSCPRCPGYAAHSLCATREDVWNGKELEGVPEEIED	IEPYVVIDDN
AT4G01930.1	309	1.15e-47	TSVLGVVKSV	CGVCHQKVDWTCGGFSCQRCHGYVVHSKCATRKDVWNGKELEGVPEETED	IQPYVVIDEN
AT4G01920.1	313	1.15e-47	TSVLGVVNSV	CGVCRKKVDWSYGGFSCQRCHGYVVHSKCATRKDVWNGKELEGLPEETED	IEPYVVIDEN
AT4G14980.1	199	1.40e-47	HLGQREEHWE	CGVCRKTVDWVYGAFKCSRCPNYAVHSRCATKKEVWDGIELEDVPEEEEE	VEEPFVVINE
AT3G46810.1	318	4.47e-47	ICHLGQGNLK	CGVCRKTVDGFYGAYSCSKCSSYVVHSLCATRKDVWDMVELEGTPEEEEI	APFEVVDENT
AT2G04500.1	137	4.47e-47	HLGQEERDWE	CGVCRRTIDWVYGAYTCSLCPSYAVHSKCATRKEVWDGRELEDVSEEDEE	IEDPFKVINE
AT1G55420.1	316	4.47e-47	TSSLQPGDFS	CGVCRQTVDVNYGQYSCNKECHYAVHSKCATRNDVWDGKDLDGVPEEPDE	FIEPPFLKID
AT5G54050.1	233	5.42e-47	HLGEKEGDWE	CGVCRKMINWVYGGYKCSRCPSYAVHSKCATKKEVWDGLELEDVPEEEEE	IKDPFKVINE
AT1G53340.1	291	5.42e-47	TSSLSSGKWS	CGVCRQEVDNDYGAYSCNKCDDYFVHSRCALRRDIWDGIELEGVPEELEI	IVEPFITISD
AT5G02360.1	136	6.55e-47	TSSLPSGKWS	CGVCRQMVDNDYGAYSCNKCDDYFVHSRCALRSDLWDGIELEGVPEEPEI	VVEPFITISD
AT5G54040.1	226	7.93e-47	HLGQGEGDWE	CGVCRKTINWVYGGYKCSRCPSYAVHSRCATRYEVWDGLELEDVPEEEEE	IEDPFKVINE
AT3G28650.1	307	7.93e-47	KYPLGPGDWI	CGVCWEEINWSYGAYSCSSCPNYAIHSRCATRKDVWDGKELDGVPELIED	IKPFKMNDDN
AT2G13900.1	309	1.16e-46	TSSLPSGKWS	CGVCRQKVDNNCGAYTCNKCNHYFVHTRCALREDVWDGKDLEGVPEEPEI	ILEPFETIAD
AT1G58037.2	205	1.16e-46	TYHLGQGDWE	CGVCRKQIDWTFGGYSCKRCPSYAVHSKCATRGDVWEGEELEDEPEEEEI	EDPYKFLLHK
AT1G58037.1	205	1.16e-46	TYHLGQGDWE	CGVCRKQIDWTFGGYSCKRCPSYAVHSKCATRGDVWEGEELEDEPEEEEI	EDPYKVIDEK
AT1G55390.1	319	1.16e-46	TSSLQVGDFS	CGVCRQTVDINYGHYSCNKGCNYAVHSKCATREDVWDGKDLEGVPEKPDE	DIEPFVRIDE
AT5G54020.1	203	1.68e-46	HLGQGEGDKE	CGVCRKKINWAYGGYECSRCPSYAVHSRCATRREVWDGLELEDVPEEEEE	IEDPFKVVNE
AT2G19660.1	307	2.45e-46	TRRLGHGQNT	CGVCRKKVDGFYGGYSCSKCSNYVVHSRCATDEDVWDMIELEGTPEEPEE	DAPFEVIDDN
AT1G55380.1	277	5.13e-46	TPSLQPGDFS	CGVCRQTVDVNYGQYSCDKECHYAVHSKCATRNDVWDGKDLDGVPEEPDE	FIEPPFLKID
AT3G06990.1	185	8.89e-46	YHLGHGDWDL	CGLCRKEIDWSFGAFTCKRCPSYAVHSKCAVRKDVWDGEELEDVPEEEEE	IEDPYKVIND
AT5G42840.2	305	1.53e-45	KYPLGPGEWR	CGVCWEEIDWSYGAYSCSLCPNYAMHSLCATRKDVWDGRELDGVLEEIED	IEPFKRNDDN
AT5G42840.1	305	1.53e-45	KYPLGPGEWR	CGVCWEEIDWSYGAYSCSLCPNYAMHSLCATRKDVWDGRELDGVLEEIED	IEPFKRNDDN
AT5G54030.1	211	5.36e-45	HLGKKEGDWE	CGVCRKMINCVYGGYKCSRCPSYAVHSRCATRKEVWDGLELEDVPEEEEE	IEDPFKVIND
AT5G45730.1	198	7.63e-45	TYHLGHGDWE	CGVCRQKMDWTLGAFFCSRCPNYAVHSKCATREDVWDGKELEDKPEDEGE	EDPYKVINEK
AT5G02350.1	307	9.09e-45	ISSLPSGKWS	CGVCRQKVNNNCGAYTCNKCRHYFVHTRCALREDVWNGKDLEGVPEEPEI	ILEPFETIAD
AT1G55440.1	269	9.09e-45	TLSLQPGDFS	CGVCRKTVDVNYGQYSCIKGCHYAVHSHCATRNDVWDGEDLEGVPEEPDE	DIEPFVRIDV
AT5G02330.1	307	1.08e-44	TSSLPSGKWS	CGVCRKKVDNNCGAYTCNKCSHYFVHTRCALREDLWDGKDLEGVPEEPEI	ILEPFETIAD
AT3G27490.1	314	1.54e-44	KYPLGPGEWR	CGVCWEEIDWSYGAYPCSLCPNYAIHSLCATRKDVWDGKELDEVPEEVED	IEPFKRNDDN
AT3G46800.1	327	2.59e-44	TSLLGHGNWT	CGVCRKKVDGFYGAYSCSKCPSYAVHARCATRKDVWYKVELEGTPEEEEI	APFEVVDENL
AT4G01910.1	310	7.23e-44	TSVLGVVNSV	CEVCHRKVDWTCGGFSCQRCSGYVVHSKCATNKDVWNGKELEGVPEETED	IKPFVVIDNN
AT1G50190.1	217	1.20e-43	FAVPSGDELS	CGVCRKLVDVNYGQYACKKGCHYAVHSKCATKNEVWDGKDLDGVPEEEEE	EIEPFVRIDE
AT5G40320.1	234	1.99e-43	TYFLGSGDWK	CGVCRRRMDGRYGAYSCSICPDYAVHSRCATRRDVWDGLELEDEPEDIEE	CEEPFRVVSD
AT3G45530.1	307	2.35e-43	KYPLGLGEWR	CGVCFEEIDWSCGAYSCSLCPQYAIHSLCATRKDVWNGKELDGVPEEVED	IEPFKRNDDN
AT3G27500.1	309	2.35e-43	KYPLGPGDWR	CGVCWDEIDWSYGAYSCSLCSRYAIHSLCATRDDVWDGEELDGVPEEIED	IEPFKRNDDN
AT2G28460.1	354	2.35e-43	TPSFDEGDWS	CSVCRKKIDNDYGGYVCTKGCSYAAHSKCATQSNVWDGIELEGEPEDIEE	EVLPPFLEIS
AT1G61840.1	467	3.29e-43	TASLQSEEWS	CGVCRKSIDGDYGAYTCEKCKDYVVHVRCALRKDVWDGVELEGVPEEDDI	TKDVGPFEII
AT2G19650.1	340	4.58e-43	TPRLGHGDGK	CRVCRKNVDGFYGAYSCSKCFDYFVHSRCATRRDVWDMIELEGTPEEEET	APFKVIDDNT
AT2G13950.1	197	1.04e-42	TSSLPYGKWS	CGVCRREVDNHYGAYSCIKCGDYFVHTRCALRKDVWNGEDLEGVPEEPEI	DVEPYETIAD
AT5G55770.1	319	1.23e-42	TSSLPSRKLS	CGVCRKQVDNKYGAYSCLECDAYFVHSSCATHPKVWDGKELEGVPEEDDI	IDDGEPFERI
AT1G44020.1	464	1.44e-42	TSFDEGDWSC	GVCRRKIDNDYGGFSCIKDGCSYAAHSRCATQKNVWDGIDLEGVPEEIEE	EEVEPFVTIS
AT2G02620.1	167	1.70e-42	TSSLPLGIWS	CGVCRKEIDGDYGAYICNICSGYAVHTRCALRLDIWDGIDLEGVPEEDEE	VEPFERISDG
AT5G55800.1	254	2.76e-42	TSSLLSGTFS	CGVCREQVDNNYGAYACNKCDGYFVHSKCAIDPKVWDGKELEGVPEENDK	IDDGEPFKRI
AT1G44050.1	363	2.76e-42	TSSLPSRTLS	CGVCHQQVDNNYGAYSCNNCDGYFVHSKCAIHPKVWDGKELEGVPEEDDL	IDDGEPFERI
AT4G10370.1	295	3.81e-42	TSSLPCGKLS	CGVCHQQVDNDYGAYSCKKCDAYFVHSKCALQRHVWDGKDLEEVPEEDDM	IDDGEPFKRI
AT3G43890.1	318	5.24e-42	TSLIGVVNSV	CGICRKKMDWSCGGYSCQKCSSYVFHTKCATREDVWDGKEMKDEPEEEEA	IQPFTIIDEN
AT1G62030.1	353	9.90e-42	HSFGQGDWSC	CVCRRKIDNDYGGYSCIKEGCSYVAHSKCATQKNVWDGKELEGILEEDEK	VEPPFVRISD
AT2G02640.1	283	2.17e-41	ISSLPFEEKS	CGVCRQMVDSDYGAYTCNVCSGYVVHVRCALRKDIWDGKELAEVPEELED	VESFRRLADG
AT2G02610.1	283	2.17e-41	ISSLPFEEKS	CGVCRQMVDSDYGAYTCNVCSGYVVHVRCALRKDIWDGKELAEVPEELED	VESFRKIANG
AT3G21210.1	444	3.47e-41	NSSLTPEQWL	CGVCRESIDTNFARYSCVKGCSFAVHSKCATRKDVWDGKELEGEPEEDES	LYIFKSFNEI
AT5G02340.1	275	4.74e-41	TSSLPSAKWS	CGVCRGEVDNHYGAYSCNKCGDYFVHTRCALRKDVWNGEDLEGIPEEPEM	DVEPYETITD
AT3G26550.1	288	7.52e-41	ASSLPYGNLS	CGVCHQRVDNNYGAYSCGKCDAYFVHSKCAFHRNVWDGKELEGVSEEDDI	IDDGEPFERI
AT2G23100.1	336	8.77e-41	ISSLISGDFS	CGVCRKTIDISYGHYSCIKGCCYGAHSKCATNKNVWDGKDLEDVPEEPEE	VIEPFVRIDE
AT4G15070.1	582	1.39e-40	LSFDQGVRCC	GVCRRKIDNDYGGYSCKKDGCSYVAHSKCATQSNVWDGKELEGEPEEDIN	EIEPFVTLRD
AT2G04680.1	313	1.39e-40	TRRLGHGEWK	CKVCRKKVDGFYGAYTCLKCPSFVVHSLCATRNDVWDMIELEGMQEEEEI	APYEMIDANT
AT1G44030.1	484	1.62e-40	TSFDEGDWSC	GVCRKTIDNDYGGYSCIKDGCSYAAHSRCATQKNVWDGIYLEGVPEEIEE	EEVEPFVTIS
AT5G48320.1	616	1.88e-40	PSFDQEVRCC	GVCRRKIDNDYGGYSCNKDGCWYAAHSKCATQSNVWDGKELEGEPEEDIK	KIEPFVTVRD
AT4G26380.1	676	1.88e-40	FSFDRGDLSC	SVCRRKIDEDYGGYTCIKDGCSYAAHSKCATQKNVWDGIELEGEPEEVEE	EVVDPFVTIS
AT2G43220.1	192	4.01e-40	SYPLKRGNWV	CEICRQDINTTYGGYSCNKCPDFFVHSKCATRKDVWDGTELEGIPEEVVD	VAPFEVIEEG
AT3G27510.1	63	4.66e-40	KYPLGPGEWR	CGVCWEEIDWSYGAYSCSVCPRYAIHSLCATRRDVWDGKELNRVPEKVEY	IEPFKRNDDN
AT1G55430.1	293	7.29e-40	TSSLQPGDFS	CGLCRHTVDVNCGQYSCDKGCQYAIHSKCATWRDVWDGIDLDGVPEEPEE	YIEPPFLKID
AT2G02700.1	294	1.14e-39	TSSLPSGKWC	CGICRREIDSDYGAYSCNVCKDYAVHTRCALRKDIWDGIELEGVPEDKDV	ELPFYRIADG
AT2G02680.1	302	1.14e-39	TSSLLKGKWS	CGVCRQEVDRKYGAYTCNACNGYTVHTRCALRNDIWDGKELEGVQEDEEV	EPPFKRISDG
AT2G02630.1	89	1.32e-39	ISSLPFEEKS	CGVCRQMLDSDYGAYICKGCSGFVAHIRCALRSDIWDGKELEGVPEEVEE	DDVESFKRIA
AT5G29624.1	221	1.53e-39	HLGEREGGWE	CGVCQTMINWVYGAYKCSRCPSYVVHSGCATKKEVWDGLDLEDVPDEEEE	IKDPFKVINE
AT1G55700.1	309	1.53e-39	SPSLPSGIYP	CGVCRLTIDVTYGQFSCNRGCHYAVHSKCAIEERIWDGKDLDGVPEEPDE	DVEPFERIDD
AT2G02690.1	306	2.38e-39	TSSLPLEKWS	CGVCRREVDHDYGAYTCCVCSGYAVHTRCALRTDIWDGIELEGVQEDEDV	EPPFKGIAGG
AT5G03360.2	347	3.69e-39	HSFEKGDWSC	GVCRKKIDNDYGGYSCIKDGCFYGAHSRCATRRSVWDGKELEGEPEENEE	ELKPFVRISD
AT5G03360.1	347	3.69e-39	HSFEKGDWSC	GVCRKKIDNDYGGYSCIKDGCFYGAHSRCATRRSVWDGKELEGEPEENEE	ELKPFVRISD
AT3G50010.1	404	3.69e-39	SSFEKEGLSC	GVCRRKINKLYGGYSCIKNGCWYAAHSRCATQKNVWDGVELEGVPEENEE	EVDPPFATKG
AT1G34480.1	248	1.36e-38	ASSFLPRKWF	CGVCRKKVDENYGGYSCVKGCYYVSHSRCALHKDVWDGKELEEEPDEIYK	DILPFKEIAG
AT3G48400.1	307	1.57e-38	THHLGLQYSE	CGVCRQSVNGYYGGYSCSLCPNYVVHPQCAMKDDVWDGIELEGTPEDSEQ	DTAPFEVVDD
AT4G02540.2	466	1.81e-38	SFDPEKYWSC	GVCRRKIENDYGCYSCVKDGCSYAAHSKCATQTNVWDGKELEGEPEEDMD	EEVESFVTIS
AT4G02540.1	466	1.81e-38	SFDPEKYWSC	GVCRRKIENDYGCYSCVKDGCSYAAHSKCATQTNVWDGKELEGEPEEDMD	EEVESFVTIS
AT2G40050.2	266	4.23e-38	TFSLTSREWF	CGVCRLSINVDYGAYTCDTCSDYVVHSICALGKDVWDGKELEGIPEIIDI	TENDGPFEVI
AT2G40050.1	266	4.23e-38	TFSLTSREWF	CGVCRLSINVDYGAYTCDTCSDYVVHSICALGKDVWDGKELEGIPEIIDI	TENDGPFEVI
AT5G37210.1	347	6.46e-38	PSFHQGNWFC	GVCRRKIENDFGGYSCVKYGCSYAAHSRCATQSNVWDGVELEGEPEEDIE	EIPPFVRISD
AT1G65180.1	305	6.46e-38	TSLLGIIDSV	CGVCRKKMDWSCGGYSCKRCSESTFHTKCATREDVWDGIEMKDEPEEDEP	MEPFEVISEG
AT5G55780.1	299	8.55e-38	TSSLPPGNFS	CGVCRQQVDNNYGAYSCEICDDYFVHSKCSLLPRIWDGKELEGVPEEDDK	IDDGEPFKRI
AT4G01350.1	305	8.55e-38	THHLGTGYSK	CGVCHENVNQYKGAYSCSVCPHYAVHSTCAVRTDVWDGVELEGTTEITED	NSPFKVVGDN
AT1G69150.1	278	8.55e-38	TSLIGVVNSV	CGICRKNMDWSCGGYSCKRCPTSVFHTKCVTREDVWDGKEMKDEPEEEED	ILPFKVIDEN
AT4G13130.1	389	1.13e-37	PSFEKGNWYC	GICRKKMDNDYGGYTCLKDGCSYVAHSRCATQKNVWDGLDLEGEPEEVEE	EEVEPFVIIS
AT5G59920.1	305	1.72e-37	THHLGTGYSE	CGVCHKNVSQYKGAYSCSVCPNYAVHSTCAVRTDVWDGVELEGTTEITED	ISPFKVVGDN
AT1G29180.1	90	2.98e-37	PLFDQRDRSC	GVCRETIDNDFGGYSCIKDDCSYGAHSRCATQRNVWDGIELEGVLEEIEE	EVEPFVTISD
AT4G10560.1	347	4.50e-37	PSFDQERLSC	GVCQKNIDNDYGGYSCIKEGCFYAAHSKCATQRNVWDGKELEGEQEEEIK	EVEPFVKISD
AT5G37620.1	316	1.34e-36	TRRIRHGKGI	CKVCRKMVDGFYGGYICVKCLSFAIHARCATREDVWEMVELEGTPEEDEI	GPFEVIDDNT

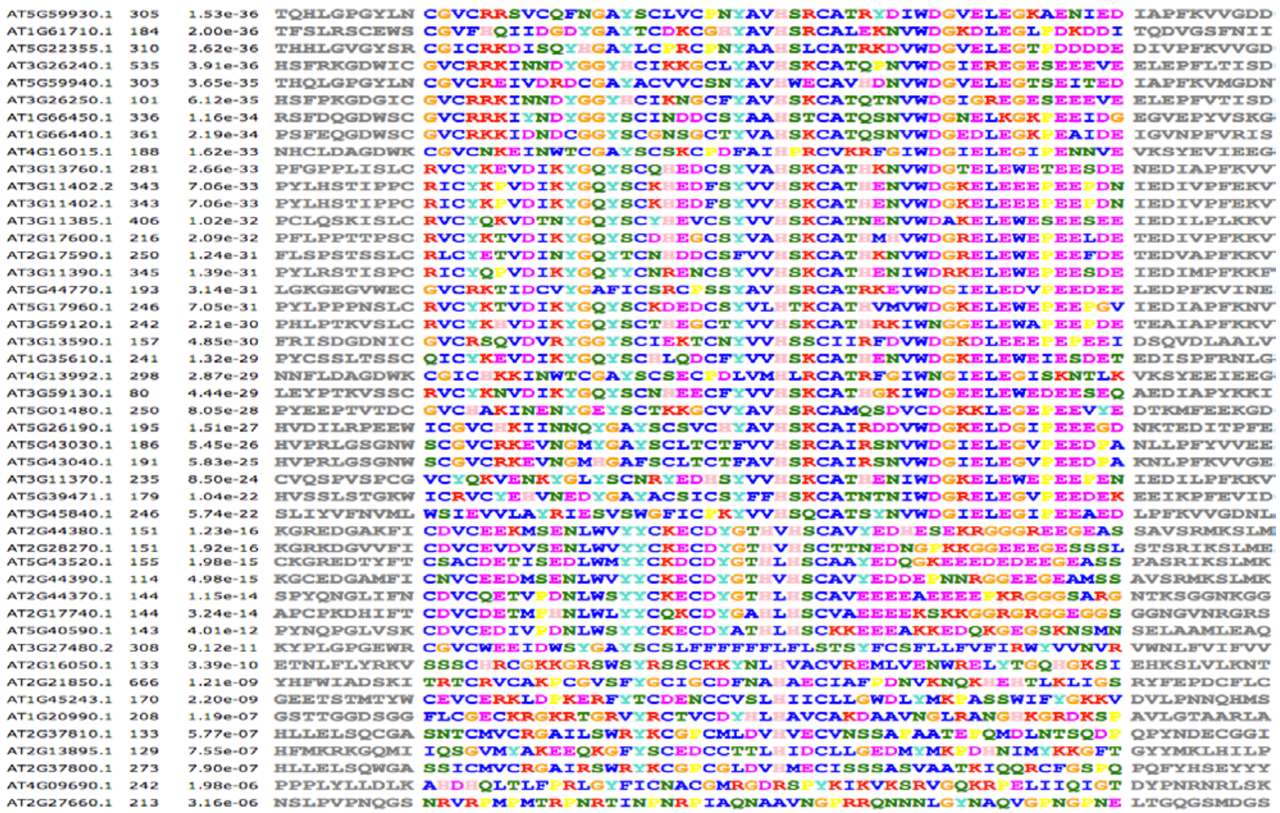

Figure S1B. Multiple sequence alignment of C1_3 domain of 73 C1-clan genes.
The multiple sequence alignment of C1_3 domain of 73 C1 clan genes in *Arabidopsis thaliana*. The gene name is followed by amino acid position, followed by P-value and the amino acids corresponding to C1_3 domain are highlighted in color.

AT4G01930.1	199	3.39e-51	GKLPDYSDRK	CRLCGRYIGDRLFYHCSSCNFTLDMRCVLNPPQQSLLNLKAHDHQLTLLPRLDSFTCNACGLKGDRSPYV	CFQCGFMIHQ
AT4G02180.1	232	3.35e-50	GQLPDNCDGK	CRLCARKIDDRLFYHCSPCNFTLDLRCVLNPPQQSLLNLKAHDHQLTLLPRLLSFTCNACGLNGDRSPYT	CVQCDFMIHQ
AT4G02190.1	204	4.05e-50	GQLPDYSDGK	CRLCEKKIDSPLFYHCSPCNFTLDMRCALNPPSISFEDSKTHDHQLTLLPRLDSFTCNACGLKGDRSPYI	CVQCNFIIHQ
AT4G01910.1	200	4.05e-50	GQRPDYSDGA	CRLCARKIDDRYFYHCSSCNFTLDMRCVLHPPQQSLLNLKAHDHQLTLLPRLDSFTCNACGLKGDRSPYV	CFQCGFMIHQ
AT4G01760.1	209	1.47e-46	GQPPDYSDGK	CRLCGRKIDDKLFYHCSSCNFTLDMRCVLNPPPKSVLDLKTHDHQLNLLPRLIFFTCNACGLNGDRSPYA	CFQCDFLIHK
AT4G11550.1	201	4.25e-44	GQPPDYSDGK	CRLCAQKICDRLFYHCSACNFSLDMCCLLNPPSQYVMDLKVHAHELTFRPRLDSFTCNACGLSGDRNPYI	CVQCDFMIHQ
AT2G13900.1	198	8.28e-44	KLYNRPGHRV	SCMCCDLALCSMLYHCPTYKLSMHPVCAMKPIPIFIDHSKRHPHLLTFFPKQASLLCDVCGLVKESFPTY	VCVRCIFVVH
AT5G55780.1	188	2.64e-43	LRLFSGSSNQ	KCSCCKVYTPNMYYHCTTCELSMNPVCAMRPVPLVVDHPKSHPHPLSFFPTQASTVCNICAMIKKLDPTY	ICIQCVFVIH
AT2G40050.2	155	2.64e-43	FFHDDNFRKI	ECLCCGRKAHWLIYHCTTCQAFMHPICAMKPIPFLIDPPKKHIHPLTFFPRQTSLTCNVCGLLRKIYPTY	VCFRCNFVTH
AT2G40050.1	155	2.64e-43	FFHDDNFRKI	ECLCCGRKAHWLIYHCTTCQAFMHPICAMKPIPFLIDPPKKHIHPLTFFPRQTSLTCNVCGLLRKIYPTY	VCFRCNFVTH
AT2G23100.1	223	8.34e-43	DNDLSDIYYG	KCKCCQKRFEDIYYHCSVCNFSLNFTCTIKPPPLTITHLKSHSHILTLFPRRIPLPCDVCGLSLNDAHDP	VYACLPCNYM
AT5G02350.1	196	9.82e-43	KLYHNFSENV	NCICCDLALFSMLYHSSTYKLSMHPVCAMKPIPILIDHPKRHPHLLTFFPKQASLLCDVCGLVKESFPTY	VCVRCIFVAH
AT5G45730.1	88	2.21e-42	SPETIDYTDG	KCQFCRSPLVDLMYHCSICNFSVDLRCWLNPAPLTIYEQKSHSHTLTLMARKDAFTCNACGLVGDRNPYV	CLECDFMLHK
AT3G26550.1	177	3.60e-42	LRLYSHPTHM	VCICCGRLVSNMFYHCVTCDLSMDPICAMEPIPFVVDHPKSHPHPITFFPTQATLACNICGLVKMLDPTY	ICIQCVFVIH
AT1G55700.1	196	4.96e-42	VSSDSSISDL	KCKCCQTNLQEVYYHCSICKFNLNLTCARNPPLSTISHLKSHEHPLSIFPRRIPLPCDACGLSLENTNDY	VYACLSCNLM
AT2G28460.1	243	8.03e-42	VLLDESSYTR	VCYCCDEDLKRMFYYCRTCDFGLNFVCAKKEAILYIDQPKWHEHTLALFLRKTSLTCNACGLSHSSCPLY	MCPPCDFVIH
AT4G10370.1	184	2.09e-41	LRLYYHPAPE	FCICCKTEVFMIFYHCLTCNLSMHPVCAMRKVPFFIDHPKSHPHPLTFFPTQASLVCHFCALIKKLDPTY	ICTKCVFVIH
AT4G02540.2	346	3.94e-41	VSLQMLSSTR	KCYCCDEDLKNIFYYCSACEFAVNFACVQKPPELSMHHPKWHEHTLALFPTQTPFPCSICALTHSSGPFY	TSSGPFYTCP
AT4G02540.1	346	3.94e-41	VSLQMLSSTR	KCYCCDEDLKNIFYYCSACEFAVNFACVQKPPELSMHHPKWHEHTLALFPTQTPFPCSICALTHSSGPFY	TSSGPFYTCP
AT4G26380.1	564	6.33e-41	LSQSSGGRTR	ECYCCDEDLLHIFYCCLACNFSMNVACTKKPAVLYRNHPRWHEHTLALFPRQASLTCNLCALADSSSPIY	MCPPCDFVVH
AT2G02620.1	56	6.33e-41	QIYLSTKPFE	HCILCSRKAHNIIYYCDVCDIYMHVLCAQATIPFFIDQPKRHDHTLTLFPRQASLTCNVCGLVNKLHLTY	VCSICNFAAH
AT5G22355.1	200	8.67e-41	YESLTSDAEE	TCLLCGERPDKVLYRCSICNFSVCRFCTKDPPPLAIEHHKTHEHRLVLLSRLISFECNACGMQGDRSPYM	CVQCGFVVHR
AT1G53340.1	180	8.67e-41	QLYSPPSGRV	LCSCCQKPIYGMNYYCPTSNFTLHLFCAFKPIPFVIDHPKRHPHPLTFFPKQSFLPCHVCSLIKKFIPTY	ICIRCAFVVH
AT5G02330.1	196	2.22e-40	KLYTRHEQLM	SCMCCKHPSNLIFYYCPTYKLSLHPDCAMKPISIFIDHPKRHPHLLTFFPKQASLLCDVCSLIKEAFPTY	VCLRCIFVAH
AT1G55390.1	206	3.02e-40	ASISDGNDHR	KCKCCQDPLKKVHYHCSICKFNLNLSCSMRPPPPTISHLKSHEHTLTLFPIRLPSPCDACGLSLSDTIDL	IYACLPCSHM
AT5G40320.1	124	3.53e-40	HGLPQEAEDD	KCRLCGEKVGKLVYHCSICDFSLDLFCARNPLSLVVYFRKGHEHTLTLMPRLIRFTCNACGLESDRSPYI	CAQCDFMIHE
AT1G65180.1	195	1.21e-39	GKPPAYTDGN	CSLCGKKIFDEMFYHCSACNFTLDLRCVSLPPPLNLHDQNTHNHELTLMPKLISFTCTTCGLHGDRSPYV	CLQCNFTSHN
AT3G07000.1	90	1.41e-39	SPGTIDYTDG	KCHFCREELVDPMYHCSLCNFSIDVNCWRHPPQRTIYQPKSHEHTFTLMPRKITFTCNACGMLGDCNPYF	CFECGFMLHK
AT3G59120.1	130	3.54e-39	IGSKSNIIFD	KCTLCGNVLSEWFYRCSICNFFLDFRCARNFPPLTIQNPRSHHHSLALFRRPVSFPCDACGLINLLDPSY	ACHQCDYMVH
AT2G43220.1	82	5.57e-39	LQGPPSYSDE	KCSLCQRKLSNFVYHCKLCNFSIDMECGNRPPPHKVDHPKCHEHALTLMGREVSFTCNACGTHGERNPYV	CLPCSVMFHY
AT2G02690.1	194	5.57e-39	LCFSSLRGVH	QCIICKNRADLIIYYCALCDIYMHAVCAQAAIPFFIDQPKRHDHTLSLFPRQASLTCNVCALVNKLHLTY	VCRSICDFVA
AT1G44050.1	252	6.14e-38	LRLCSFQTRL	TSGCCRKITFGMFYQCTTCNLSMHPVCAMRPVPLVVDHPKSHPHPLSFFPTQASTVCHICAKIKHLDPTY	ICIQCVFVIH
AT2G13950.1	86	1.11e-37	QLYSSPFALV	NCICCDISFQDIIYHSSNHKLSMHPVCAMKPVPIFIDHTKRHPHPLTFSPREAFLPCNVCGLIKESILTY	VCVPCDFVVH
AT1G61840.1	356	1.73e-37	SYRLPSAPDI	ECLCCGTTATDLVYGCTICEAVMHTPCAMKSIPFVIDQPKSHDHSLTLFPRQASLTCNVCGLLRKNYPTF	VCLRCNFVAH
AT1G55420.1	203	2.00e-37	EASLSDGNHL	KCKCCGVPLQMTYYHCSICKFNLNLDCSMRQPPPIISHLKSHEHTLTLFPIRLPLPCDACGLSLNDTEDL	VYSCLPCSRM
AT5G59920.1	195	2.68e-37	ESLTDDAEKS	CLLCGNIPAENMLYHCSVCNFTSCLGCTKNPPLLVIEHMKTHKHPLTLLPRRISCICDVCGKKCQFTAYV	CLQCDFVTAR
AT3G46810.1	208	5.56e-37	YADHKCLLCG	REFEDQQGQKYELYHCDVCNFSICISCQGNPPPLVVVTPKTHEHQLHLIPRLLDFTCNACGTQGDRSPYF	CLQCNFMIHR
AT1G66450.1	224	8.60e-37	LWWVSNFQKR	KCYCCDEELLWIFYFCSACDCGMNIACVEKKPLLSIDHPEWHEHILSLFPRQAPLPCSVCALPHSRSTFY	ICSPCQFIVH
AT1G55430.1	179	8.60e-37	GASLSYGNHL	KCKCCASPLEKLYYHCSTCKFNLNLTCSMRPPPTTISHLKFHEHTLTLFLVRITWPCDVCGFLLNKDTDD	LVYACLPCSL
AT2G02700.1	182	1.77e-36	LQLYNVKTFE	HCILSTKKAEVLLYYCALCDIYMHVLCAQAKIPFLIDQPKKHDHTLTLFPRQASLTCNVCGLVDKLHLTY	VCRSICDFVA
AT5G55800.1	143	1.31e-35	LRLTSSPQSQ	NCICCHHYFNDIFYHCSTCKLIMHPICAMRSIPFAVDHPKSHSHPLTFYPAQASLVCHFCASIKKFDPTY	ICIQCVFAIH
AT4G01350.1	195	1.74e-35	ESLTDDAETT	CLLCGKKPAENMLYHCSVCNFTSCLGCTKNPPLLVIEHIKTHKHPLILFPRRIPCICDICGKKCEFAVYV	CPQCDFVTAR
AT5G01480.1	135	4.04e-35	DMSLEEERDK	RCCSCKRGVDALFYYCSICDFAVHMACAGNPTSPTIDNPKRHKHTLHYFPRKSGLFCDVCALVDDSDYLY	VCLLCDFIVH
AT2G02680.1	191	1.63e-34	QLYSSKKRFK	YCIMCRKKVNGLVYYCALCDMYMHTLCAQAVIPFFIDQPKRHAHILTLFPRQASLTCNICGVLDELHFTY	VCPICDFVTH
AT3G27473.1	196	[illegible]e-34	GAPDYTEDRS	CHICGKETGNLLYHCDICKFNLDMECAVENPIPVALSDLKVHEHTLTLMPRLISFICDACGTKGDRAPYV	CVQCDFRMFH
AT1G55440.1	156	4.27e-34	DASLSDGNHR	SCRCCNKPLDKTYYHCSICKFNLNLPCSMMLPLLTISHMKSHEHTLTLFCTRLPMSCDACGLSLSTRSTL	LYTCLPCSHT
AT3G43890.1	208	7.38e-34	EPPAYTDGKC	RLCGEKLANFEVFYHCSACNFSLDLQCVFHPPKQNPHDLNIHDHPLTLMPKSISFTCTTCGLNGDRSPYV	CLPCDFTSHN
AT3G21210.1	333	7.38e-34	HALRFRYESG	QICLSCSTFKDFAYHCSICNFTMCQDCAREPPLFAIDYPKRHEHTLFYFQRKGSLICDVCGLSDGRSLLY	SCVKCNFVVH
AT5G59930.1	195	1.11e-33	SESLTDNDKK	TCFLCGQQPHGILYHCSICNFTLCIGCIKSPPPLVVENVKTHKHPLTFFPRGVYCTCNVCGVKGLILSYM	CLQCGFAVHC
AT2G04680.1	203	6.42e-33	APDYADNKCL	LCEEEFDQQHPKIHHCDVCNFTICRACMKNAPPVRIESLTTHEHQLHLVPRRIDFTCNACGTLGDRSPYF	CLQCNFMIHR
AT3G27490.1	203	1.10e-32	DGAPNYTDPR	CHICGEDTQHLLYHCDICKFNLDLRCAAKSPPPIALSNLKVHEHTLTLMPRLISFVCDACGTEGDRSPYI	CVQCDFMIFH
AT3G11385.1	294	1.87e-32	KKLGSTESDH	CTWCGKSIQGSWSYRCSICNFCLDLSCSQNIPLLLVPNPKSHQHPLVFYRRPLLTPCDACGLINMLDPSY	ACFQCNYMVH
AT3G13760.1	169	4.70e-32	SKSDIIFDKC	TWCGKDFKGDVWFYRCLICGFCLDLSCALTLPPLTIANPKSHHHSLLFLPRPLLVPCDACGLVNALEPSY	ACFQCNYMIH
AT5G42840.2	194	6.12e-32	NGAPDYTNLN	CHICGDATGNLLYHCDICKFNLNMRCAIREPTPVALSNMKVHEHTLTLMPRLISFVCDACGMKGDRAPYV	CVQCDFMIFH
AT5G42840.1	194	6.12e-32	NGAPDYTNLN	CHICGDATGNLLYHCDICKFNLNMRCAIREPTPVALSNMKVHEHTLTLMPRLISFVCDACGMKGDRAPYV	CVQCDFMIFH
AT5G37620.1	206	1.03e-31	EAPDYADKKC	LLCDEKFDQDQGLHHCDVCNFSICRACMKNPPPVGVESPTTHEHQLHLVPRRIHFTCNACGTLGEQSPYF	CLQCNFMIHR
AT5G44770.1	80	1.74e-31	FGQFGEDDDY	QCHYCQQNVGFHFARCTICNISIDEKCLRNPPPLTIFQPKHHKHSLFLLGRLVAFTCNACGVEGDRNPYI	CIECNLMLHK
AT1G35610.1	129	4.27e-30	PYKSDIIFNK	CTWCAKDFKGDWFYRCLICSFCLDLSCAATLPLLTITNPKSHHHSLVFLPRPLLVPCDACGLVDGLEPSY	ACFQCNYMVH
AT3G45530.1	197	1.17e-29	EGAPTYTDPK	CHLCGEDTGKFIYHCDICKFNLDLVCAKTKTPPVALSNLKVHEHTLNLMPRLISFVCDACGTKGDRAPYV	CHQCDFMVHK
AT2G04500.1	24	4.03e-29	KIFINGNHDC	HFCKIDLSTTSLYARCTICNVNMDLDCMVNLPPLTIFEPKHHKHSFNLLLRVVTFTCNACGLEGDRNPYV	CYECHLMVHK
AT5G17960.1	134	6.60e-29	FDNCTWCGKY	IPTPSSHPRMTVFYRCSICNFCLDTMCTQTKPPLTIENPKGHHHSLVLFPRPLLVPCDACGLVNASEASY	ACFQCNYVVH
AT3G27500.1	198	1.08e-28	SGAPNYTNPR	CHICGEDTGNLLYHCDICKFNLDLQCAIKSSPPVALSNMKVHEHTLTLMPKLISFVCDACGTKGDRSPYI	CVQCDFMTFH
AT1G34480.1	137	1.08e-28	VLVSDYLKLN	TICYCCCRRKELNFYCSICNFCICANCEAKPPLLTIDHKKRHEHMLSYFPREASITCNVCALDERRYFLY	ICHQCDFVVH
AT5G55770.1	209	8.02e-27	LRLYGLPTFQ	KCFCCETSLYDMFYHCATCDLSMSPVCALKPVPIVIEQSRSHHHPLTFFPAQALICHICAVIKKFDPAYI	CVQCVFVVHK
AT3G28650.1	196	1.69e-23	DGAPDYTDPK	CHICGVETANLLYHCDMCMFNLDIGCAIRNPERVSLSKLKVHEHTLTLMPKLMFFVCDACGMKGDHAPYV	CVQCDFMFFH
AT4G13130.1	277	1.16e-22	QWSEEETMIE	CYCCDEYLMDYYYYCSSCDFAMNVSCLEKPPPVLSIDHPKWHQHPLILFPSQASFPCNLCALTHSSCPFY	ICPPCDFVVH

AT3G46800.1	217	1.15e-21	AHEKCLLCKK	KFKKLSNNYDRVYHCDVCNVTICKDYCMAKPPPVSVVSPTTHEHQLHLVPRLVNFTCNACGTAGDRSPYF	CLQCNFMIHR
AT5G59940.1	81	2.44e-17	FLTNGLGDLP	CDLCGQKRLEAAGYSCPTCEFKLDLTCGINPSPPAIEHPICHDHPLVFLKKREEKAPCEVCKDSIGGPSY	SCLGCDLYFH
AT1G57820.2	14	5.84e-15	IQLPCDGDGV	CMRCKSNPPPEESLTCGTCVTPWHVSCLSSPPKTLASTLQWHCPDCSGEIDPLPVSGGATGFESAGSDLV	AAIRAIEADE
AT1G57820.1	14	5.84e-15	IQLPCDGDGV	CMRCKSNPPPEESLTCGTCVTPWHVSCLSSPPKTLASTLQWHCPDCSGEIDPLPVSGGATGFESAGSDLV	AAIRAIEADE
AT1G57800.1	14	2.01e-14	TQYPCDPEGV	CMRCKSMPPPEESLTCGTCVTPWHVSCLLSPPETLSATLQWLCPDCSGETNPLPVSGVAAGYGSVGSDLV	AAIHSIEADE
AT3G50010.1	290	6.75e-14	LVVLQEDSTR	ECYCCDEALSRILYYCSACDFAISILCARKPPVFLINHHRPEWHGHPLALFPRQAFLTCNLCALADPSSP	IYMCPPCDFV
AT1G16710.2	1169	2.04e-12	FGMYVQEFGS	ECSNPNQRRVYLSYLDSVKYFRPDIKSANGEALRTFVYHEILIGYLEYCKLRGFTSCYIWACPPLKGEDY	ILYCHPEIQK
AT1G16710.1	1198	2.04e-12	FGMYVQEFGS	ECSNPNQRRVYLSYLDSVKYFRPDIKSANGEALRTFVYHEILIGYLEYCKLRGFTSCYIWACPPLKGEDY	ILYCHPEIQK
AT5G60410.5	174	2.20e-12	CRLTRADPFW	VTVAHPLSPVRLTATTIPNDGASTMQSVERTFQITRADKDLLAKPEYDVQAWCMLLNDKVLFRMQWPQYA	DLQVNGVPVR
AT5G60410.4	174	2.20e-12	CRLTRADPFW	VTVAHPLSPVRLTATTIPNDGASTMQSVERTFQITRADKDLLAKPEYDVQAWCMLLNDKVLFRMQWPQYA	DLQVNGVPVR
AT5G60410.3	174	2.20e-12	CRLTRADPFW	VTVAHPLSPVRLTATTIPNDGASTMQSVERTFQITRADKDLLAKPEYDVQAWCMLLNDKVLFRMQWPQYA	DLQVNGVPVR
AT5G60410.2	174	2.20e-12	CRLTRADPFW	VTVAHPLSPVRLTATTIPNDGASTMQSVERTFQITRADKDLLAKPEYDVQAWCMLLNDKVLFRMQWPQYA	DLQVNGVPVR
AT5G60410.1	174	2.20e-12	CRLTRADPFW	VTVAHPLSPVRLTATTIPNDGASTMQSVERTFQITRADKDLLAKPEYDVQAWCMLLNDKVLFRMQWPQYA	DLQVNGVPVR
AT5G26190.1	86	2.20e-12	ERIIGYRRRD	QCCLCRYKLLDMFYNCSRCMFEVCMACLKRPLLIYKTTSHKHPLSLMPIDSVSFTCDACGSVDNSSYPCT	CLQCCFIIHR
AT2G21830.1	21	5.75e-12	PLTSPEIVAE	GICNICFQDQVVEYACKPCNFDICKTCSKLPQKVSHGFHSDHPLEFSLFQYDRKPGYIVCSCCGNMSSCS	FYECKECEIY
AT3G12980.1	1169	2.44e-11	FGMYVQEFGT	DSASPNQRRVYLSYLDSVKYFRPDVRTVSGEALRTFVYHEILIGYLDYCKKRGFSSCYIWACPPLKGEDY	ILYCHPEIQK
AT1G66050.1	14	3.74e-11	TQLPCDGDGV	CMRCQVNPPSEETLTCGTCVTPWHVSCLLPESLASSTGDWECPDCSGVVVPSAAPGTGISGPESSGSVLV	TAIRAIQADV
AT1G66040.1	14	4.95e-11	TQLPCDGDGV	CMRCQVTPPSEETLTCGTCVTPWHVSCLLPESLASSTGDWECPDCSGVVVPSAAPGTGISGPESSGSVLV	AAIRAIQADV
AT2G02470.2	82	6.54e-11	EPALGINFAR	DGMQEKDWVSLVAVHSDSWLLSVAFYFGARFGFGKNERKRLFQMINELPTIFEVVSGNAKQSKDLSVNNN	NSKSKPSGVK
AT2G02470.1	82	6.54e-11	EPALGINFAR	DGMQEKDWVSLVAVHSDSWLLSVAFYFGARFGFGKNERKRLFQMINELPTIFEVVSGNAKQSKDLSVNNN	NSKSKPSGVK
AT2G37800.1	34	1.14e-10	GPEQTHQNRR	MCDICDESAEGLYYQCKPCGFDVHPLCTQLPQHSWRYKCGQCRVDVHMECVDSSTSVAETGIQQRGFGQQ	FEFPQYHQPY
AT3G45840.1	199	1.22e-10	SKSLPDDAEK	ACVLCGVRSEIVMYYCLICDFSMCLNCVRSPPPLTAKSLIYVFNVMLWSIEVVLAYRIESVSWGFICPKY	VVHSQCATSY
AT1G67220.1	888	1.60e-10	FALFVQEFGS	ECSQPNQRSTYIFYLDSVKYFKPERVTFAGEALRTFVYHEVLIGYLEYCKLRGFTTSYIWACPPKIGQDY	IMYSHPKTQQ
AT3G27510.1	256	1.71e-10	YLKQRFGDVK	TCQSCGMDETEVVIGCMKCDYFLDFRCATLPLTVTLPRYDDHPLTLCYGEEKASGKYWCDICERETNPKT	WFYTCKDCGI
AT1G14510.1	82	2.94e-10	EPALGINFAR	DGMQEKDWISLVAVHSDSWLISVAFYFGARFGFGKNERKRLFQMINDLPTIFEVVTGNAKQSKDQSANHN	SSRSKSSGGK
AT2G31650.1	664	3.60e-10	NLCRPGAPDM	PPRCCLCPVVGGAMKPTTDGRWAHLACAIWIPETCLSDVKKMEPIDGVNKVSKDRWKLMCTICGVSYGAC	IQCSNNSCRV
AT3G13590.1	45	3.85e-10	VHIFEESPRD	SRDAICKLCKSTVSFESFAYYCRECRSHFHKDCLTIINAKIHEHTLTFIRRENPFPCDVCGRDDKGMDMY	GCLQCDFFVH
AT3G42790.1	83	4.11e-10	EPALGINFAR	DGLSEKEWLSLVAIHSDAWLLSVSFYFGSRFSFHKEERKRLFNMINDVPTIFEVVTGMAKAKDKSSAANQ	NGNKSKSNSK
AT1G77800.2	341	4.40e-10	ADSERPCLLC	PKKGGILKPVLSKTENGGPAEFAHLFCSLWMPEVYIEDLKKMEPILNFPGIKETRRKLLCNLCKVKSGAC	IRCCNVVVLT
AT1G77800.1	341	4.40e-10	ADSERPCLLC	PKKGGILKPVLSKTENGGPAEFAHLFCSLWMPEVYIEDLKKMEPILNFPGIKETRRKLLCNLCKVKSGAC	IRCCNVVVLT
AT1G05830.2	681	5.02e-10	NLCRPVALDI	PPRCCLCPVVGGAMKPTTDGRWAHLACAIWIPETCLLDVKKMEPIDGVKKVSKDRWKLLCSICGVSYGAC	IQCSNNTCRV
AT1G05830.1	681	5.02e-10	NLCRPVALDI	PPRCCLCPVVGGAMKPTTDGRWAHLACAIWIPETCLLDVKKMEPIDGVKKVSKDRWKLLCSICGVSYGAC	IQCSNNTCRV
AT4G23860.3	115	5.73e-10	KLLPSKDIEN	SENSYNHNFKGLYCTCDRPYPDPNVEEQVEMIQCCLCEDWFHEEHLGLTPSDSVGSQIPRDEESEPIYED	FICQNCSPAC
AT4G23860.2	115	5.73e-10	KLLPSKDIEN	SENSYNHNFKGLYCTCDRPYPDPNVEEQVEMIQCCLCEDWFHEEHLGLTPSDSVGSQIPRDEESEPIYED	FICQNCSPAC
AT4G23860.1	115	5.73e-10	KLLPSKDIEN	SENSYNHNFKGLYCTCDRPYPDPNVEEQVEMIQCCLCEDWFHEEHLGLTPSDSVGSQIPRDEESEPIYED	FICQNCSPAC
AT5G39550.1	14	1.18e-09	TQLPCDGDGV	CMRCQVNPPSEETLTCGTCVTPWHVPCLLPESLASSTGEWECPDCSGVVVPSAAPGTGNARPESSGSVLV	AAIRAIQADE
AT2G21850.1	21	1.18e-09	PLTSPKIAVD	GICSICYKDDLVELACSPCDFSLCKGCSQLPQKVWHDFHPDHPLEFCLHQYDQKPEHVVCSGCGNMTSGS	FYKCKECEIY
AT5G48160.2	141	2.24e-09	AQSVDEFMFL	QKVVQSRTDLSSVTLVRAHRVQLEILVAINTGIQAFLHPNISLSQPSLIEIFVYKRCRNIACQNQLPADD	CYCDICTNRK
AT5G48160.1	141	2.24e-09	AQSVDEFMFL	QKVVQSRTDLSSVTLVRAHRVQLEILVAINTGIQAFLHPNISLSQPSLIEIFVYKRCRNIACQNQLPADD	CYCDICTNRK
AT5G26210.1	82	3.95e-09	EPVLGINFAR	DGMAEKDWLSLVAVHSDAWLLAVAFFFGARFGFDKADRKRLFNMVNDLPTIFEVVAGTAKKQGKDKSSVS	NNSSNRSKSS
AT5G35210.2	1326	4.48e-09	QGNSGLDSDS	ERMSEQKDSKPSTPLPATPLYPPDDVFIPEDDPLLVSVSKVKQITPSSFDLEWSTTAFAPGPQKLPVRRQ	VKREDSDAAY
AT5G35210.1	1326	4.48e-09	QGNSGLDSDS	ERMSEQKDSKPSTPLPATPLYPPDDVFIPEDDPLLVSVSKVKQITPSSFDLEWSTTAFAPGPQKLPVRRQ	VKREDSDAAY
AT5G36740.1	735	6.12e-09	HYPHTLADHQ	ACINQDGTVPGERSTDSFCGKYCQELFEELQLFIGVKHPLPEGFSWSFLRRFELPSEVADCDISEKIAYN	AKMAVAFSVM
AT5G36670.1	735	6.12e-09	HYPHTLADHQ	ACINQDGTVPGERSTDSFCGKYCQELFEELQLFIGVKHPLPEGFSWSFLRRFELPSEVADCDISEKIAYN	AKMAVAFSVM
AT2G21840.1	21	1.53e-08	ELTSRGIVAN	GICNICFKDEPVEFACDPCNFDLCKACSDLPQKMSHHLHPEHPLEFRIGEDDQKTKHMVCVVCGNMSSGA	KYYYECSKCE
AT5G53430.1	289	2.07e-08	WVRRGMIFPF	VDYVERLQEQSELRGCNPRDFQMALEEALLADQGFTEKLMQDIHMAAGNQTFDDSVYRWVEEAAGSSQYL	DHVAPSQDMK
AT3G51120.1	1117	2.19e-08	SILDNVSAGK	SSMLTEAKDNTPFSHCGTAGPELLLFAPPPPPPTAISHDLTLTTTALRLGSETTVEAGTVERLPKSVLGV	SSEPSPRSLS
AT5G05610.2	164	2.95e-08	NGVKRSIEGQ	TKSTPKLMEESYEDEDDEHGDTLCGSCGGNYTNDEFWICCDVCERWYHGKCVKITPAKAESIKQYKCPSC	CTKKGRQ
AT5G05610.1	164	2.95e-08	NGVKRSIEGQ	TKSTPKLMEESYEDEDDEHGDTLCGSCGGNYTNDEFWICCDVCERWYHGKCVKITPAKAESIKQYKCPSC	CTKKGRQ
AT5G24330.1	144	3.13e-08	LLPYNPSNDP	QRRLEQMASLATALRASNTKFSNELTYVSGKAPRSANQAAFEKGGMQVLSKEGVETLALCKKMMDLGECP	PLMVVFDPYE
AT5G09790.2	175	3.32e-08	LLPLVPSEDP	DQRLAQMGTLASALTALGIKYSDGLNYVPGMAPRSANQSKLEKGGMQVLCKEDLETLEQCQSMYRRGECP	PLVVVFDPLE
AT5G09790.1	148	3.32e-08	LLPLVPSEDP	DQRLAQMGTLASALTALGIKYSDGLNYVPGMAPRSANQSKLEKGGMQVLCKEDLETLEQCQSMYRRGECP	PLVVVFDPLE
AT3G20280.2	374	3.96e-08	QESSKDAKVD	SEACQNHPTASPATVVPDQDSTITAAPSVTQEDSAFNTEKTPPQPLSVSSNYDSQTEKETPNVQDSVHNV	PGDSEKGKGL
AT3G20280.1	635	3.96e-08	QESSKDAKVD	SEACQNHPTASPATVVPDQDSTITAAPSVTQEDSAFNTEKTPPQPLSVSSNYDSQTEKETPNVQDSVHNV	PGDSEKGKGL
AT3G02890.1	688	3.96e-08	DLPVAAPNVL	STTSAIPKPEYIWQGDLEVQKSRNLSAMHSGIQAYLSTLASPKVVEVVKQFPEKVTLNEVPRLSSWPAQF	QDTGAKEQHV
AT5G22260.1	123	5.30e-08	KKYHFVIPSK	ETMAAFLKLEGGGYAFPEKESFSHLVELQGHVLHGFFHSNGFGHLLSLNGIETGSDLTGHQVMDLWDRLC	TGLKARKIGL
AT2G37520.1	694	5.95e-08	GRDLIPVMVY	GRNISGQEFGGMYCLVLIVNSLVVSAALLRIFGQEVAELPIVATSREYQGRGYFQGLYACVENLLSSLNV	ENLVLPAAEE
AT5G55390.2	561	6.67e-08	ISLGERLFSY	TQEPNPVKPGRVIPVDSKHNKTDSIASKEPGSEIPTLDNDSQRRLLAVMKKATEEITMGTILKKFKIQST	MSTHSTRNVV
AT5G55390.1	561	6.67e-08	ISLGERLFSY	TQEPNPVKPGRVIPVDSKHNKTDSIASKEPGSEIPTLDNDSQRRLLAVMKKATEEITMGTILKKFKIQST	MSTHSTRNVV
AT3G06990.1	437	6.67e-08	AGNTDGPLWC	DICEEETDPSVWFYGCDDCVSTLHIKCVLGDMYHFKPGNKYGKAELVANDGMTRPFCIVCEERCMFPSFL	KATVSDSTLV
AT1G63490.1	304	7.48e-08	SVFACLEHWK	HLCECEPTKLRLEYRYTLAELDMMVQEVEKFGGCKTQETKISQRPSSGTKRSIALNKKQEGMQVSQARPA	DKWLLRASKV
AT3G07780.1	140	8.39e-08	AQPIDEFMFL	QKFVQTRSDLTSKTLVRAHRVQLEVLVVINTGIQAFLHPNINLSQSSLIEIFVYKRCRNIACQNELPADG	CPCEICANRK
AT5G15540.2	1587	1.05e-07	LVYSINRVMQ	IRAGAVESNLKALLHKDSAKTQHGNGAYQQDPIPGHMNMMDLNTRIQEEPRHWNSYGHATLIDLNGSVYQ	DSRDQFTSYQ
AT5G15540.1	1589	1.05e-07	LVYSINRVMQ	IRAGAVESNLKALLHKDSAKTQHGNGAYQQDPIPGHMNMMDLNTRIQEEPRHWNSYGHATLIDLNGSVYQ	DSRDQFTSYQ
AT1G43770.2	198	1.05e-07	TNVVEPVEVS	SSSPTKETMESKRQESSDSRKPHELTCLVGDSETANSSSVPEHNSCAVKKRKLSSGNKQIQLADGSSSCR	VAESNMPLTL
AT1G43770.1	198	1.05e-07	TNVVEPVEVS	SSSPTKETMESKRQESSDSRKPHELTCLVGDSETANSSSVPEHNSCAVKKRKLSSGNKQIQLADGSSSCR	VAESNMPLTL

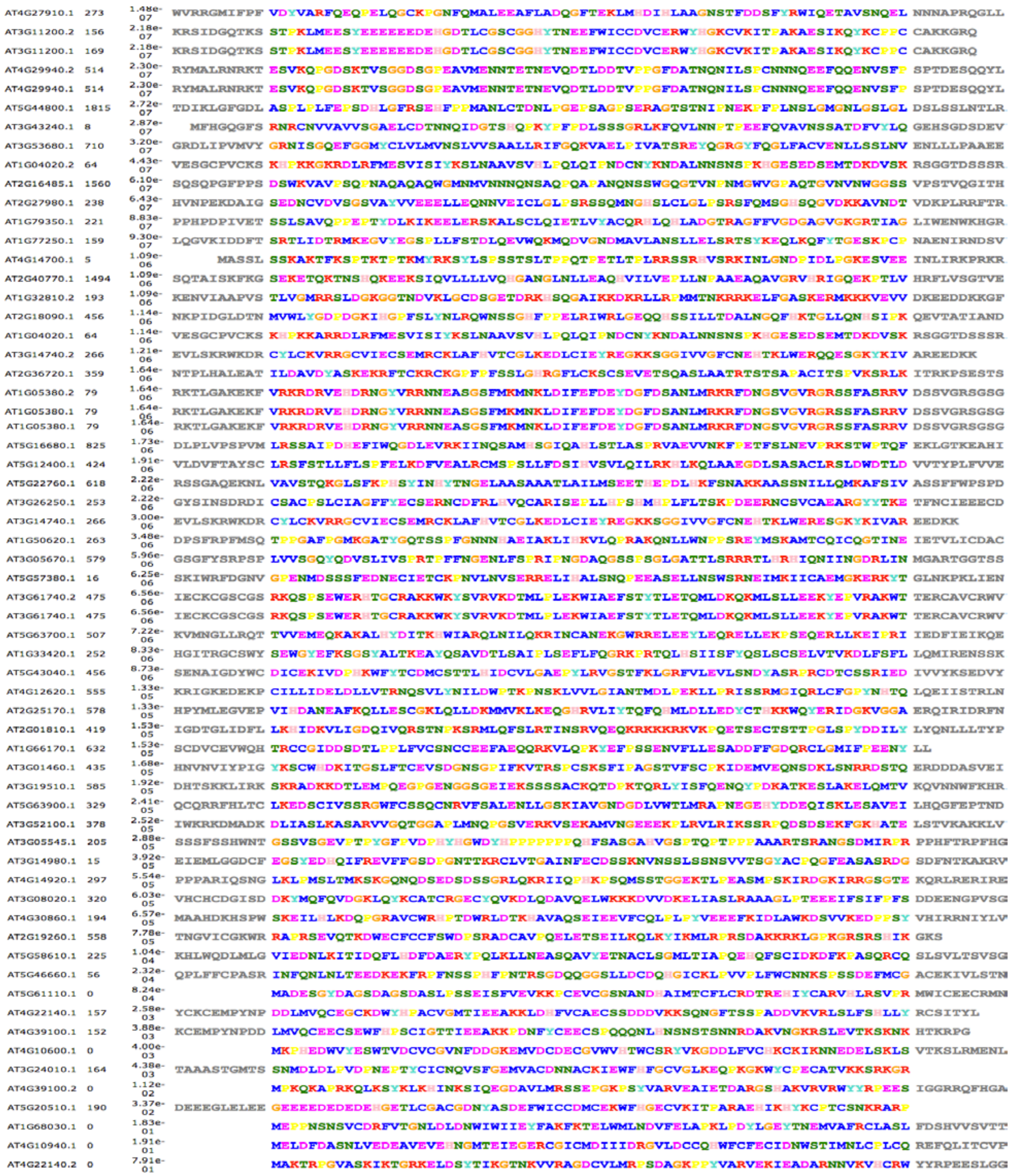

Figure S1C. Multiple sequence alignment of ZZ/PHD type domain of 73 C1-clan genes.

The multiple sequence alignment of ZZ/PHD type domain of 73 C1 clan genes in *Arabidopsis thaliana*. The gene name is followed by amino acid position, followed by P-value and the amino acids corresponding to ZZ/PHD type domain are highlighted in color.
