# Supplementary material for: A Hormone-Responsive C1-Domain-Containing Protein At5g17960 Mediates Stress Response in Arabidopsis thaliana
Source: PLoS One. 2015 Jan 15;10(1):e0115418. doi: 10.1371/journal.pone.0115418 (PMC4295845; doi:10.1371/journal.pone.0115418)
Supplement: S2 Fig — (A) The C1_2 consensus sequence is from amino acid 1–29, in total 29aa. (B) The C1_3 the consensus sequence is from amino acid 26–52, in total 27aa. (C) The ZZ/PHD type the consensus sequence is from amino acid 2–60, in total 59aa. The derived consensus sequence is shown below each diagram. X-axis refers to position of amino acid. Y-axis depicts the degree of consensus based on the size of amino acid i.e. the larger the size of amino acid symbol the more conserved it is. (PDF) [file pone.0115418.s006.pdf]

Supporting Information (Ravindran Vijay Bhaskar et al.)

Figure S2

A

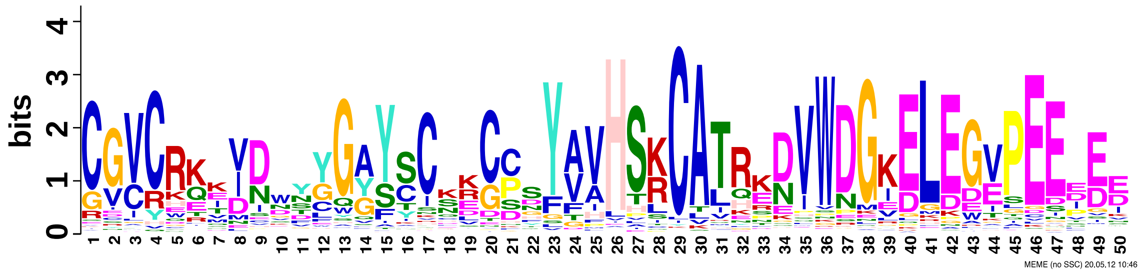

C1_2 consensus sequence: $CX_2CX_{12}CX_2CX_8C$

B

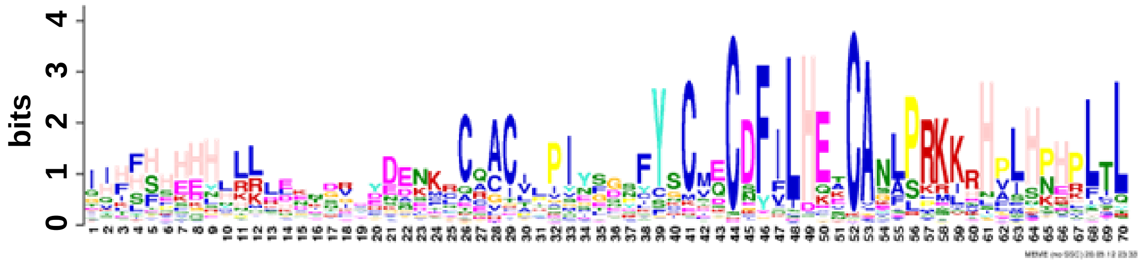

C1_3 consensus sequence: $CX_2CX_{11}CX_2CX_7C$

C

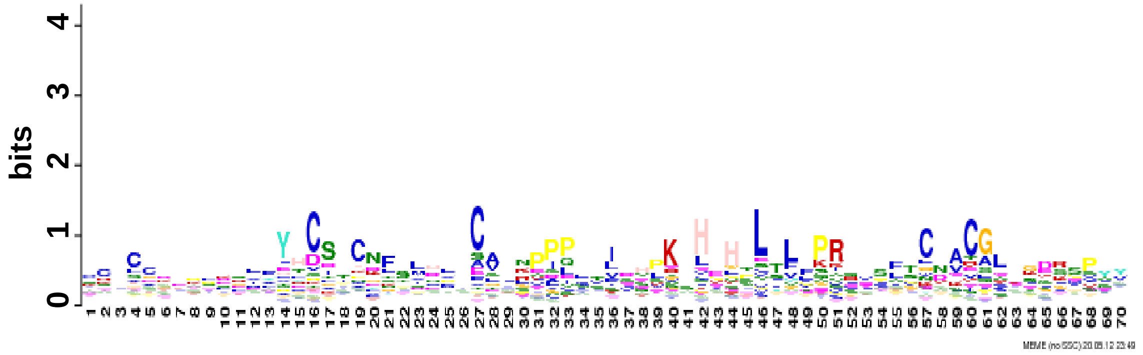

ZZ/PHD type consensus sequence: $CX_2CX_{11}CX_2CX_7CX_{28}CX_2C$

Figure S2. Consensus sequence of C1_2, C1_3 and ZZ/PHD type domains.
(A) The C1_2 consensus sequence is from amino acid 1-29, in total 29aa. (B) The C1_3 the consensus sequence is from amino acid 26 -52, in total 27aa. (C) The ZZ/PHD type the consensus sequence is from amino acid 2-60, in total 59aa. The derived consensus sequence is shown below each diagram. X-axis refers to position of amino acid. Y-axis depicts the degree of consensus based on the size of amino acid i.e. the larger the size of amino acid symbol the more conserved it is.
