# Supplementary material for: A Hormone-Responsive C1-Domain-Containing Protein At5g17960 Mediates Stress Response in Arabidopsis thaliana
Source: PLoS One. 2015 Jan 15;10(1):e0115418. doi: 10.1371/journal.pone.0115418 (PMC4295845; doi:10.1371/journal.pone.0115418)
Supplement: S3 Fig — The 73 genes were mapped to the five chromosomes of A. thaliana. The green bars represent the A. thaliana chromosomes (numbered one to five at the top of each bar). The 73 genes are represented by their accession numbers. Chromosome mapping was performed using the TAIR online Chromosome map tool. (PDF) [file pone.0115418.s007.pdf]

Supporting Information (Ravindran Vijay Bhaskar et al.)

Figure S3

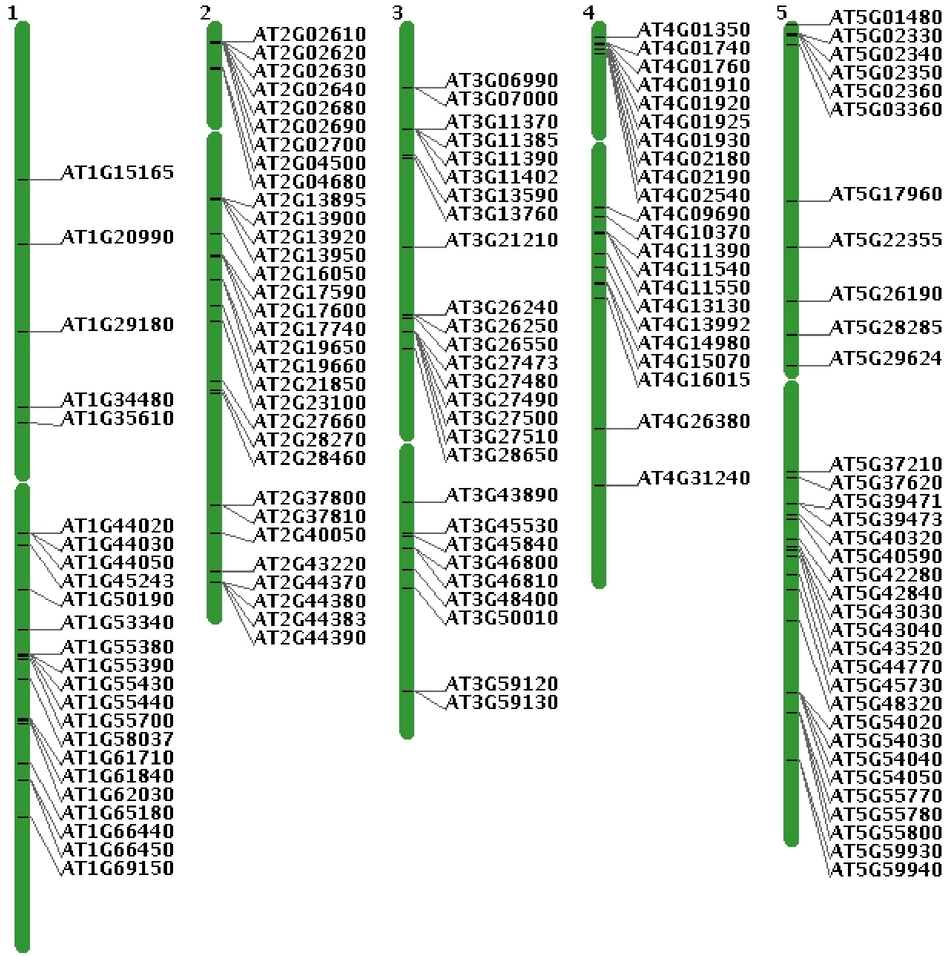

Figure S3. Location of 73 C1-clan genes on *A. thaliana* chromosomes.

The 73 genes were mapped to the five chromosomes of *A. thaliana*. The green bars represent the *A. thaliana* chromosomes (numbered one to five at the top of each bar). The 73 genes are represented by their accession numbers. Chromosome mapping was performed using the TAIR online Chromosome map tool.
