# Supplementary material for: A Hormone-Responsive C1-Domain-Containing Protein At5g17960 Mediates Stress Response in Arabidopsis thaliana
Source: PLoS One. 2015 Jan 15;10(1):e0115418. doi: 10.1371/journal.pone.0115418 (PMC4295845; doi:10.1371/journal.pone.0115418)
Supplement: S4 Fig — (A) Transcript levels of At5g17960 were significantly upregulated in the leaves of 7 selected 35S-At5g17960 independent lines in the T3 generation. (B) Transcript levels of At5g17960 were significantly decreased in the leaves of 8 selected amiRNA-At5g17960 independent lines in the T3 generation. Transcript levels in (A) and (B) were determined by qRT-PCR and are shown relative to TUB2 expression. Values are the mean ± standard deviation of three independent biological replicates each with three technical replicates. (PDF) [file pone.0115418.s008.pdf]

Supporting Information (Ravindran Vijay Bhaskar et al.)

Figure S4

A

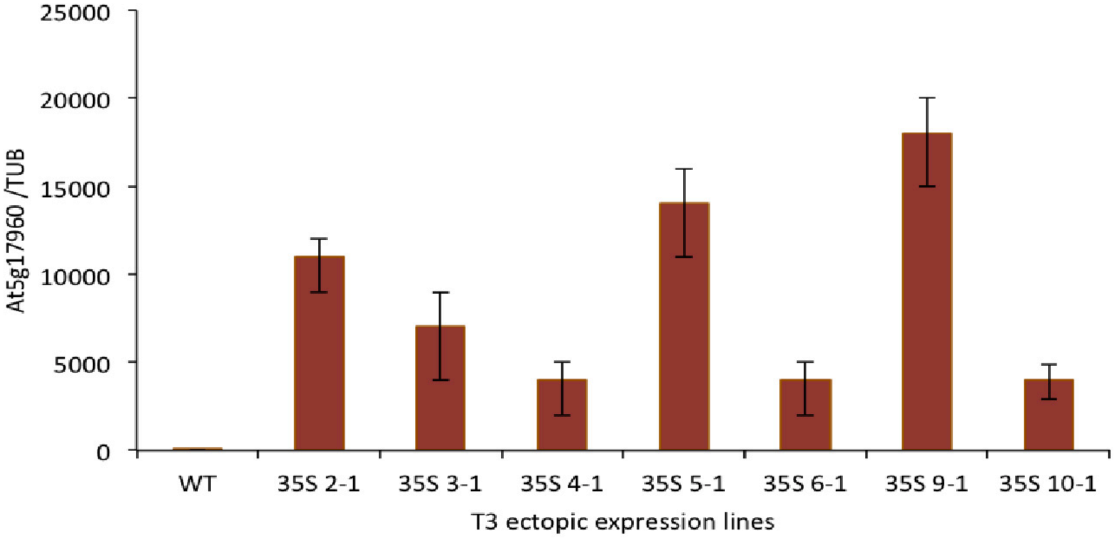

B

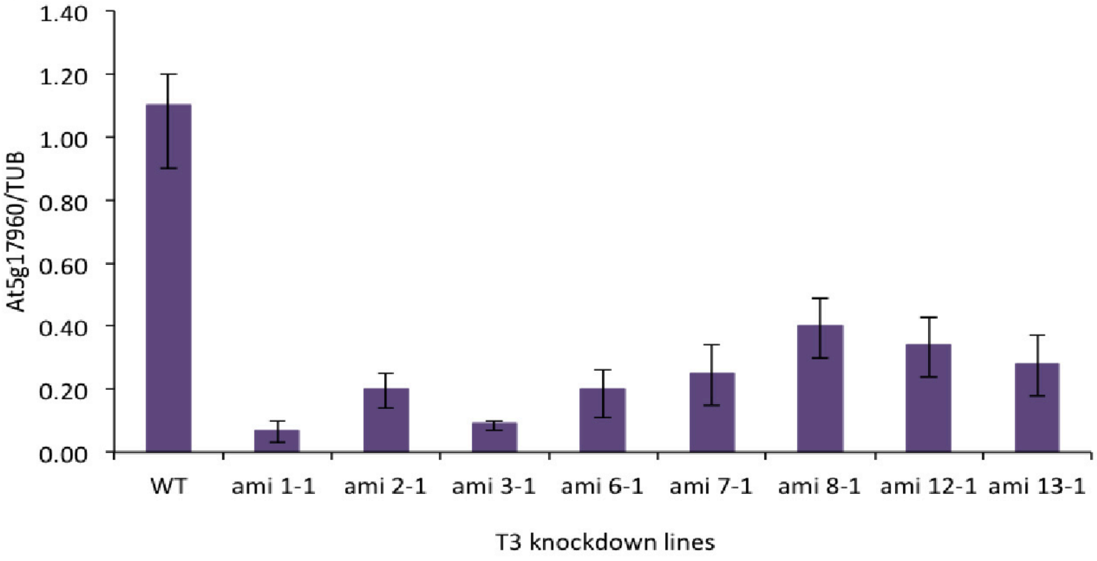

Figure S4. Expression of *At5g17960* transcripts in transgenic plants.
(A) Transcript levels of *At5g17960* were significantly upregulated in the leaves of 7 selected *35S-At5g17960* independent lines in the T3 generation. (B) Transcript levels of *At5g17960* were significantly decreased in the leaves of 8 selected *amiRNA-At5g17960* independent lines in the T3 generation. Transcript levels in (A) and (B) were determined by qRT-PCR and are shown relative to *TUB2* expression. Values are the mean ± standard deviation of three independent biological replicates each with three technical replicates.
